# Methylglyoxal Accumulation is Associated with Brain Inflammation after Myocardial Infarction with Sex and Regional Differences

**DOI:** 10.1002/advs.202522584

**Published:** 2026-04-09

**Authors:** Ramis Ileri, Xixi Guo, Erik J. Suuronen

**Affiliations:** ^1^ Bioengineering and Therapeutic Solutions (BEaTS) Program University of Ottawa Heart Institute Ottawa Canada; ^2^ Ottawa‐Carleton Institute For Biomedical Engineering (OCIBME) Ottawa Canada; ^3^ Department of Cellular and Molecular Medicine University of Ottawa Ottawa Canada

**Keywords:** heart‐brain interaction, methylglyoxal, myocardial infarction, sex differences

## Abstract

Patients with myocardial infarction (MI) have an increased risk of developing neurological disease and cognitive impairment, but the mechanisms underlying the heart‐brain interaction remain to be better elucidated. Methylglyoxal (MG), a highly reactive dicarbonyl, is a shared causative factor associated with cardiovascular and neurological diseases. MG‐derived advanced glycation end products (MG‐AGEs) accumulate in the heart and circulation post‐MI, making it a promising target in studying the heart‐brain axis. Here, we report that MG‐AGEs accumulate in the mouse brain at 6 h and 7 days post‐MI, with the highest expression observed in the brainstem, followed by the cortex. Notably, males had higher MG‐AGE expression compared to females in most brain regions. The accumulation of MG‐AGEs in the brain was correlated with increased neuroinflammation, including a greater number of activated microglia and macrophages, and increased expression of AGE receptors. Greater inflammatory factor expression (NF‐κB and TNF‐α) and a reduction in tight junction proteins of the blood brain barrier were also observed. Taken together, this study reveals a novel MG‐mediated mechanism with sex‐based differences that may contribute significantly to heart‐brain interactions after MI and identifies a promising therapeutic target for treating the neurological impairment associated with heart disease.

## Graphical abstract

This study identifies that methylglyoxal may play an important role in heart‐brain interactions after myocardial infarction. Myocardial infarction leads to increased levels of methylglyoxal‐derived advanced glycation end‐products (MG‐H1) in the brain of mice, which is associated with loss of blood‐brain barrier integrity and neuroinflammation.

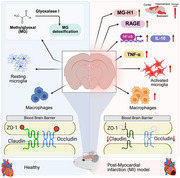

## 1 Introduction

Alterations in heart‐brain interactions are increasingly being recognized as important factors contributing to neurological and cardiovascular disease. The function of the brain can be directly affected by heart function, which is referred to as the heart‐brain axis. Myocardial infarction (MI), commonly known as a heart attack, and heart failure are leading causes of mortality worldwide [1, 2]. Clinical studies have identified that both MI and heart failure are associated with an increased risk of neurological disease (including dementia) and cognitive impairment [3, 4, 5, 6]. For example, heart attack patients are more prone to behavioral disorders such as depression compared to patients without MI [7, 8]. Additionally, it has been reported that patients with a history of MI and depression have an increased risk of future cardiovascular events [9]. However, the mechanisms whereby heart disease leads to brain injury and impaired brain health remain to be better elucidated. Understanding this link may lead to improved treatment and prevention strategies for these patients.

A potential phenomenon of interest in studying the heart‐brain axis is the post‐MI production and accumulation of methylglyoxal (MG), a highly reactive dicarbonyl compound. We previously reported that MG accumulates acutely in the heart post‐MI, and that this contributes to adverse ventricular remodeling and cardiac dysfunction [10]. MG is formed mostly as a by‐product of glycolysis, and its reaction with lipids, proteins, and DNA can lead to cell and tissue dysfunction [11]. The irreversible reaction of MG with protein generates advanced glycation end‐products (AGEs), and MG‐derived hydroimidazolone‐1 (MG‐H1) is the major product formed (∼90% of all adducts) [11, 12]. Under normal physiological conditions, MG is detoxified primarily by the glyoxalase system and the enzyme glyoxalase‐1 (Glo1) [12]. However, conditions such as inflammation, ischemia, and oxidative stress stimulate MG production, while also limiting Glo1 activity, leading to MG‐derived AGE (MG‐AGE) accumulation [13]. Ischemia, inflammation, and oxidative stress are present post‐MI, and the infarcted heart undergoes a metabolic shift to derive ATP from anaerobic glycolysis, all of which are favorable for MG formation.

Notably, in addition to increased MG in the heart, greater levels of MG and its AGEs have been reported in other tissues post‐MI. For example, clinical studies have revealed that the concentration of MG in the blood (plasma and serum) is increased in patients post‐MI [14, 15, 16]. Recently, it was shown that MG levels are elevated in the plasma of patients 24 h after MI and that the highest MG levels are associated with the lowest cardiac function at 4 days post‐MI [14]. Furthermore, AGEs have been shown to accumulate in the skin of patients after MI, and higher AGE levels were associated with inflammation, glycemic stress, and a greater incidence of major cardiac events and death at 1‐year following MI [17, 18]. These observed systemic effects suggest that the MG and its AGEs generated after MI have the potential to affect other tissues and organs, including the brain; however, this remains to be better elucidated.

**FIGURE 1:**
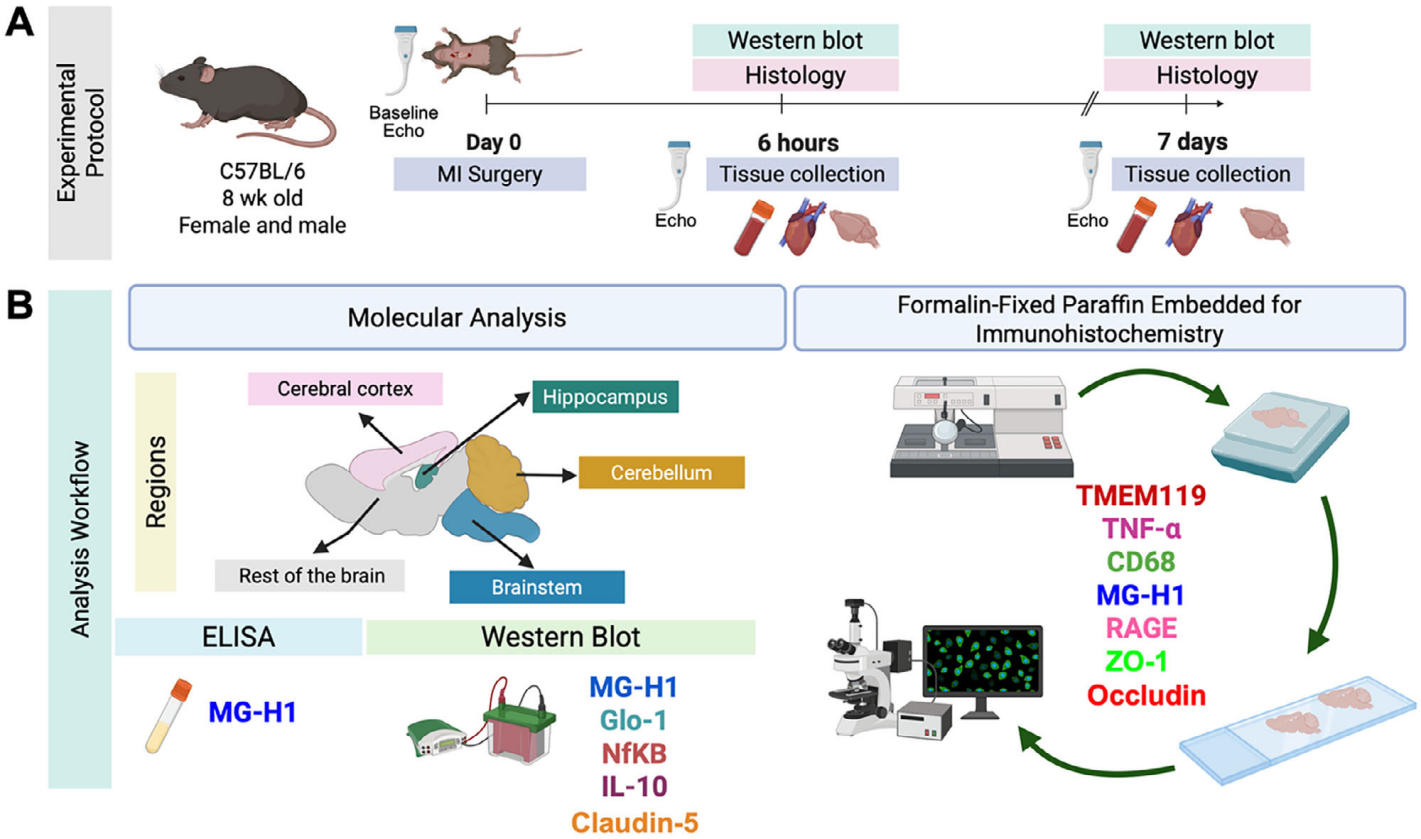
Experimental design of the study. (A) Mice underwent coronary artery ligation surgery to induce MI, and brains were collected at 6 h and 7 days post‐MI. (B) Brains were dissected into 5 different regions and prepared for molecular analysis or fixed for immunohistochemistry.

Herein, we investigated how MG may contribute to brain damage after MI. Our hypothesis was that MG‐AGEs accumulate in the brain post‐MI and that this would be associated with neuroinflammation. Coronary artery ligation surgery was performed in female and male mice to induce MI. The brains were then collected at different time‐points post‐MI, and the levels of MG‐H1, Glo1, macrophages, activated microglia, and inflammatory markers were quantified in the different brain regions (see Figure 1). We report, for the first time, that MG‐AGE levels increase in the brain after MI, and that this varies between its different regions. Furthermore, increased MG‐AGE levels were associated with greater inflammation in the brain, and females were more protected from the MG‐induced damage compared to males. This highlights a novel mechanism of MG accumulation and neuroinflammation with sex‐based differences that may play a significant role in heart‐brain interactions after MI and identifies a promising therapeutic target for mitigating neurological impairment associated with heart disease.

## 2 Results

### 2.1 MG‐H1 Expression Increases in the Brain after MI

**FIGURE 2:**
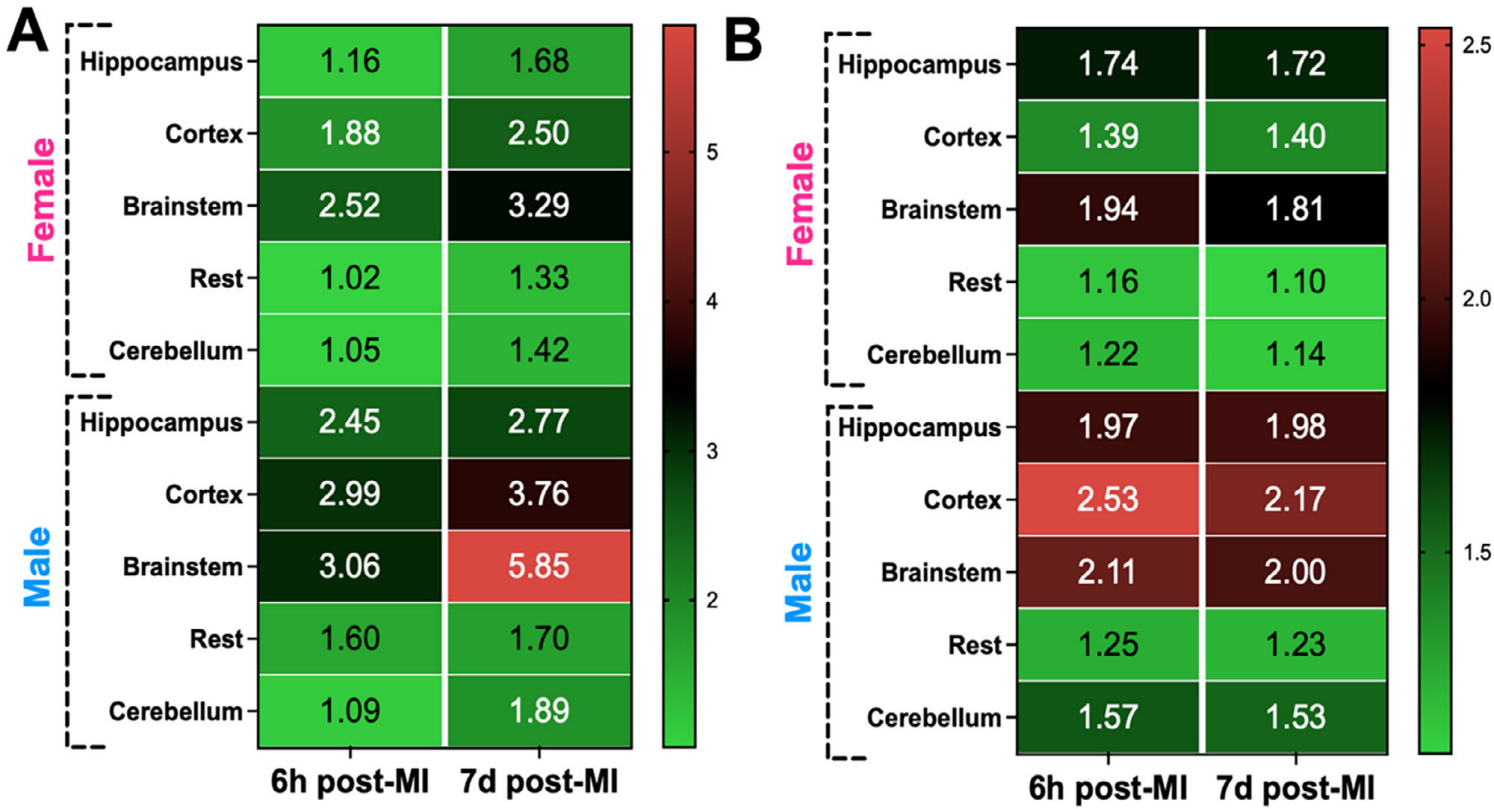
MG‐H1 accumulates in the brain after MI, with the highest levels observed in the brainstem. (A,B) Summary of fold changes for the level of MG‐H1 adducts (A) and Glo1 protein (B) in the different brain regions at 6 h and 7d post‐MI relative to the healthy group.

Coronary artery ligation surgery was performed in 8‐week‐old female and male mice to induce MI. The loss of cardiac function in female and male mice was confirmed by echocardiographic assessment of left ventricular ejection fraction at 7 days post‐MI (Figure S1A). The blood, hearts, and brains of the mice were harvested at 6 h or 7 days and prepared for molecular and immunohistochemistry analyses. Myocardial fibrosis was observed in female and male mice at 7 days post‐MI (Figure S1B,C). After dissecting the brain into the cerebral cortex, brainstem, hippocampus, cerebellum, and the rest of the brain (see Figure 1), we first determined the levels of MG‐H1 and Glo1 in the different brain regions. A summation of the Western blot results revealed that MG‐H1 was increased in multiple brain regions at 6 h and/or 7 days post‐MI compared to the healthy group, with the greatest expression observed in the brainstem (Figure 2A). While elevated MG‐H1 in the brain post‐MI was observed for both sexes, males exhibited greater increases compared to females across all brain regions (Figure 2A). The expression of the enzyme Glo1 was increased only in the cortex and brainstem of male mice at 6 h and 7 days post‐MI, with no change in expression observed in females (Figure 2B). Overall, these results suggest that MG accumulates in the brain following MI, leading to the formation and of MG‐AGEs (specifically MG‐H1), the extent of which is both region‐specific and sex‐dependent. Since MG‐H1 levels were greatest in the hippocampus, brainstem, and cortex, further analysis of these brain regions is highlighted herein, while results for the cerebellum and the rest of the brain are reported in the Supporting Information.

**FIGURE 3:**
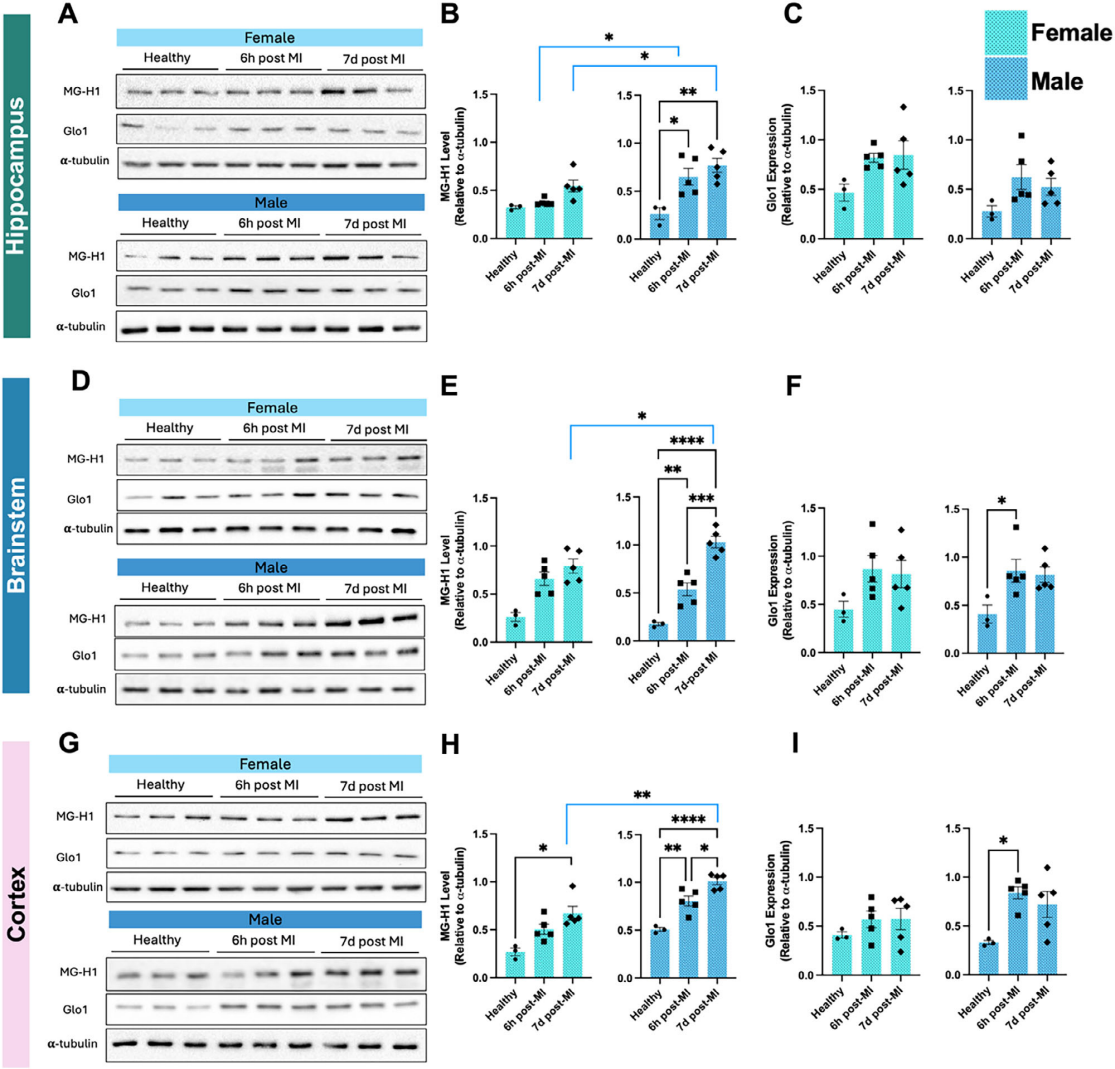
MG‐H1 levels increase in the hippocampus, brainstem, and cortex post‐MI. (A) Representative western blot images for MG‐H1 and Glo1 in the hippocampus. (B,C) Quantification of MG‐H1 (B) and Glo1 (C) relative to α‐tubulin (*n* = 3 for healthy and *n* = 5 for 6 h and 7d post‐MI groups). (D) Representative western blot images for MG‐H1 and Glo1 in the brainstem. (E,F) Quantification of MG‐H1 (E) and Glo1 (F) relative to α‐tubulin (*n* = 3 for healthy and *n* = 5 for 6 h and 7d post‐MI groups). (G) Representative western blot images for MG‐H1 and Glo1 in the cortex. (H,I) Quantification of MG‐H1 (H) and Glo1 (I) relative to α‐tubulin (*n* = 3 for healthy and *n* = 5 for 6 h and 7d post‐MI groups). Data are presented as mean ± SEM. Differences between groups were determined by a one‐way ANOVA (black line), and differences between sexes were determined by a two‐way ANOVA (blue line). ^*^ *p* < 0.05, ^**^ *p* < 0.01, ^***^ *p* < 0.001, ^****^ *p* < 0.0001.

**FIGURE 4:**
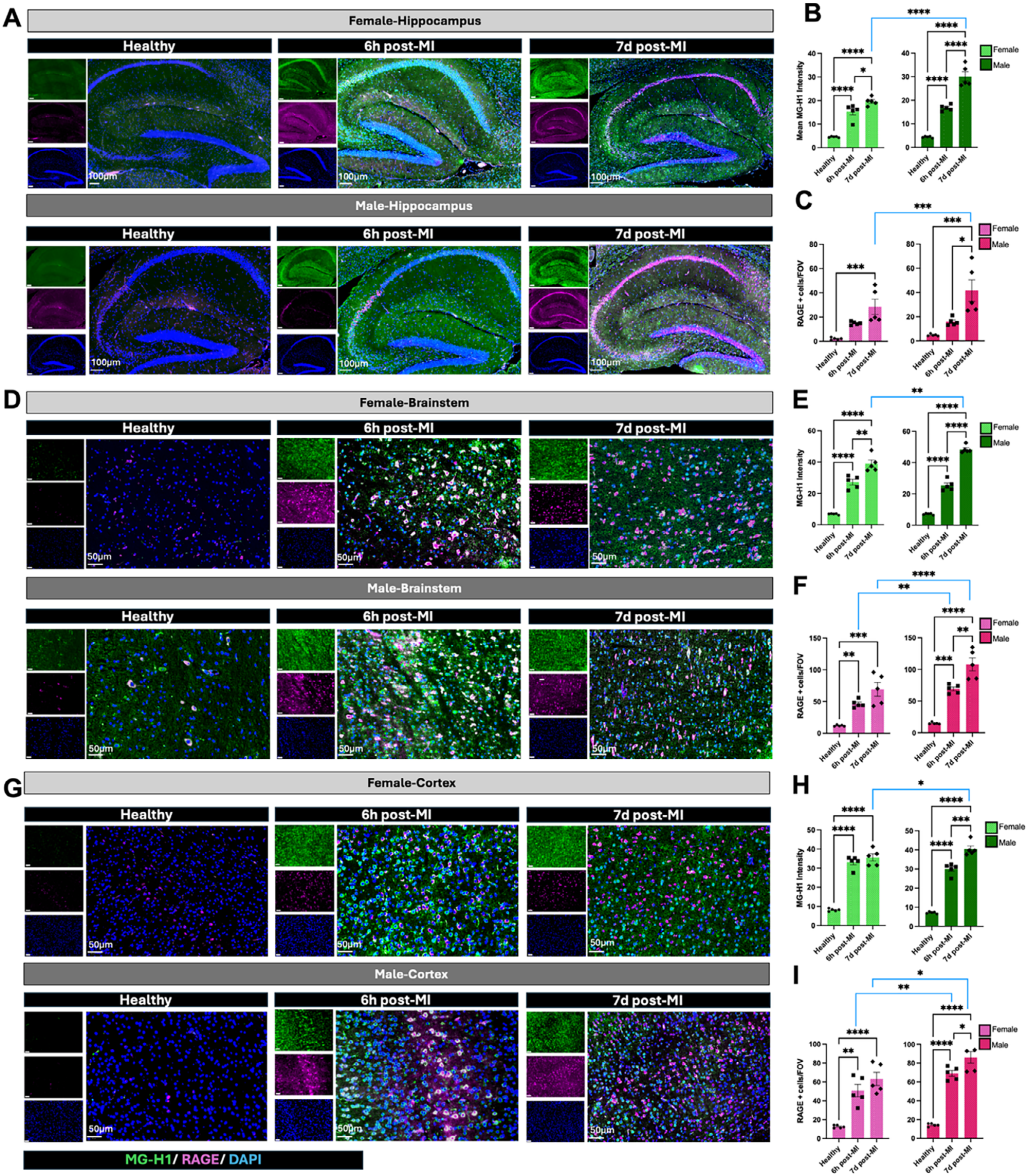
MG‐H1 and RAGE levels increase in the brain post‐MI. (A) Representative immunofluorescence images of MG‐H1 and RAGE staining in the hippocampus, scale bar = 100 µm. (B,C) Quantification of mean MG‐H1 fluorescence intensity (B) and number of RAGE^+^ cells (C) per field‐of‐view. (D) Representative immunofluorescence images of MG‐H1 and RAGE staining in the brainstem, scale bar = 50 µm. (E,F) Quantification of mean MG‐H1 fluorescence intensity (E) and number of RAGE^+^ cells (F) per field‐of‐view. (G) Representative immunofluorescence images of MG‐H1 and RAGE staining in the cortex, scale bar = 50 µm. (H,I) Quantification of mean MG‐H1 fluorescence intensity (H) and number of RAGE^+^ cells (I) per field‐of‐view. Data are presented as mean ± SEM (*n* = 5 per group). Differences between groups were determined by a one‐way ANOVA (black line), and differences between sexes were determined by a two‐way ANOVA (blue line). ^*^ *p* < 0.05, ^**^ *p* < 0.01, ^***^ *p* < 0.001, ^****^ *p* < 0.0001.

In the hippocampus, the level of MG‐H1 was increased at 6 h post‐MI in males and at 7 days post‐MI in females and males, with greater expression seen in males at both time‐points compared to females (Figure 3A,B). No change in Glo1 expression was observed in the hippocampus for males or females over this time (Figure 3A,C). In the brainstem (where the greatest MG‐H1 accumulation was observed), MG‐H1 levels at 6 h post‐MI increased by approximately 2.5‐fold in females and 3‐fold in males (Figure 3D,E). At 7 days post‐MI, these levels further increased to 3.3‐fold in females and 5.8‐fold in males relative to their respective healthy controls, with the males having significantly higher levels compared to females (Figure 3D,E). Glo1 levels more than doubled at 6 h and 7 days post‐MI in male mice, whereas no change in Glo1 was seen in female mice (Figure 3D,F). In the cortex, MG‐H1 expression increased 2.5‐fold in females and 3.8‐fold in males at 7 days post‐MI compared to the healthy group, with males exhibiting higher MG‐H1 levels compared to the females (Figure 3G,H). Glo1 levels were elevated more than 2‐fold at 6 h and 7 days post‐MI in male mice compared to healthy controls, but Glo1 did not change in female mice (Figure 3G,I). For the cerebellum and the rest of the brain, the only observed difference in MG‐H1 or Glo1 levels was an increase in MG‐H1 for male mice at 7 days post‐MI compared to the healthy group (Figure S2A–F). Immunofluorescence staining of MG‐H1 in brain tissue sections also demonstrated that the level of MG‐H1 was increased at 6 h and/or 7 days post‐MI (Figure 4A,B,D,E,G,H; Figure S3A,B,D,E). These results suggest that MG accumulates in the brain following MI, leading to the formation of MG‐AGEs, and the extent of this accumulation appears to be both region‐specific and sex‐dependent.

### 2.2 RAGE Expression is Increased in the Brain Post‐MI

AGEs bind to the receptor for AGE (RAGE) on the cell surface, and RAGE expression is upregulated in response to an increased presence of AGE [19, 20]. Moreover, RAGE activation may lead to a decrease in Glo1 expression, which, in turn, reduces MG detoxification [12, 21, 22]. Consequently, this can result in further MG‐derived AGE formation and RAGE expression, creating a vicious cycle. We examined the effect of MI on RAGE expression in the brain. Immunohistochemistry results show that the number of RAGE^+^ cells was increased at 6h and/or 7 days post‐MI in both females and males compared to the healthy controls (Figure 4A,C,D,F,G,I; Figure S3A,C,D,F). Notably, a greater number of RAGE^+^ cells was seen in males compared to females in the hippocampus at 7 days post‐MI (Figure 4C) and in the brainstem and cortex at 6 h and 7 days post‐MI (Figure 4F,I). Taken together, these findings demonstrate that an increase in MG‐H1 in the brain post‐MI is associated with greater RAGE activation.

### 2.3 Inflammation is Triggered in the Brain after MI

**FIGURE 5:**
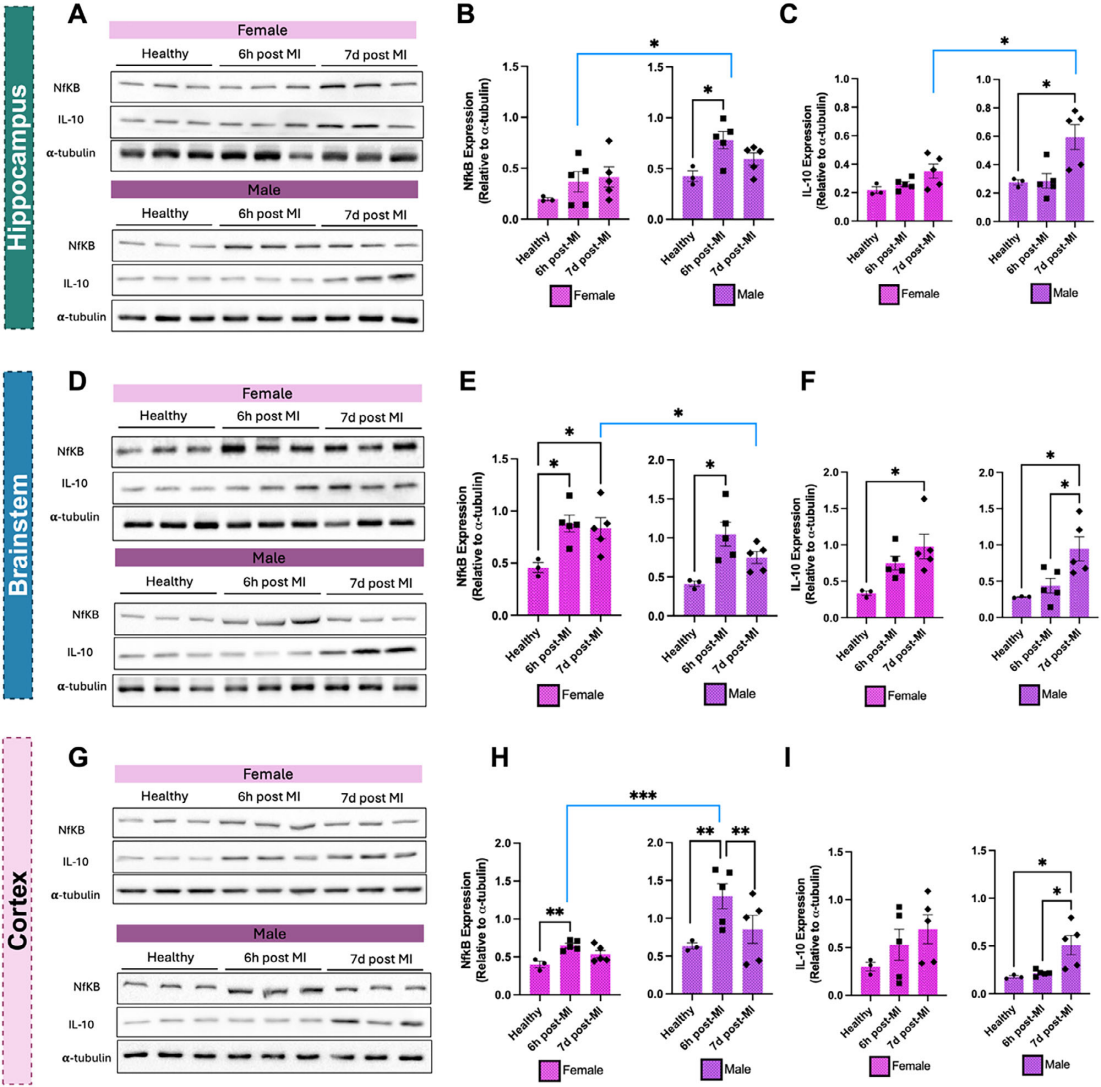
Total NF‐κB p65 and IL‐10 expression increases in the brain post‐MI. (A) Representative western blot images for total NF‐κB and IL‐10 in the hippocampus. (B,C) Quantification of total NF‐κB (B) and IL‐10 (C) relative to α‐tubulin (*n* = 3 for healthy and *n* = 5 for 6 h and 7d post‐MI groups). (D) Representative western blot images for total NF‐κB and IL‐10 in the brainstem. (E,F) Quantification of total NF‐κB (E) and IL‐10 (F) relative to α‐tubulin (*n* = 3 for healthy and *n* = 5 for 6 h and 7d post‐MI groups). (G) Representative western blot images for total NF‐κB and IL‐10 in the cortex. *Note*: these are the same blots as presented in Figure 3G, which were stripped and re‐probed. (H,I) Quantification of total NF‐κB (H) and IL‐10 (I) relative to α‐tubulin (*n* = 3 for healthy and *n* = 3–5 for 6 h and 7d post‐MI groups). Data are presented as mean ± SEM. Differences between groups were determined by a one‐way ANOVA (black line), and differences between sexes were determined by a two‐way ANOVA (blue line). ^*^ *p* < 0.05, ^**^ *p* < 0.01, ^***^ *p* < 0.001.

Since MG‐AGE accumulation and RAGE activation are known to cause inflammation, we next evaluated neuroinflammation in the brain post‐MI. A well‐known function of nuclear factor‐κB (NF‐κB) is in the regulation of inflammatory responses and the activation of inflammasomes [23]. It is also a key transcription factor involved in the differentiation of macrophages to their M1 inflammatory phenotype [24]. Thus, we measured total NF‐κB p65 expression in the different brain regions in MI mice by Western blot. Our results show that total NF‐κB expression was increased in several brain regions post‐MI in females and males, which has been summarized in Figure S4A. For females, the expression of total NF‐κB was significantly increased in the brainstem at 6 h and 7 days post‐MI and cortex at 6 h compared to the healthy control, but there was no change observed in other brain regions (Figure 5A,B,D,E,G,H; Figure S5A,B,D,E). In contrast, total NF‐κB expression in male mice was increased in the hippocampus, brainstem and in the cortex (at 6 h post‐MI), but not in other brain regions (Figure 5A,B,D,E,G,H; Figure S5A,B,D,E). In evaluating the sex‐related differences, males exhibited higher total NF‐κB expression than females in the hippocampus, cortex, and cerebellum at 6 h post‐MI, while females expressed more total NF‐κB than males in the brainstem at 7 days post‐MI.

We also examined the expression of interleukin‐10 (IL‐10), a potent anti‐inflammatory cytokine [25], in the same brain regions of MI mice, which is summarized in Figure S4B. For females, IL‐10 expression was increased in the brainstem and rest of the brain at 7 days post‐MI, while males had significantly greater IL‐10 levels at 7 days post‐MI in the hippocampus, brainstem, and cortex compared to healthy mice (Figure 5A,C,D,F,G,I; Figure S5A,C,D,F). Elevated IL‐10 likely reflects compensatory anti‐inflammatory signaling in response to inflammatory activation. Comparing the sexes, males had greater IL‐10 expression than females in the hippocampus at 7 days post‐MI, but there were no differences observed in the other brain regions. These total NF‐κB and IL‐10 results indicate that there is an increase in both pro‐ and anti‐inflammatory cytokine signaling in the brain after MI.

### 2.4 MI Leads to Microglial Activation in the Brain

**FIGURE 6:**
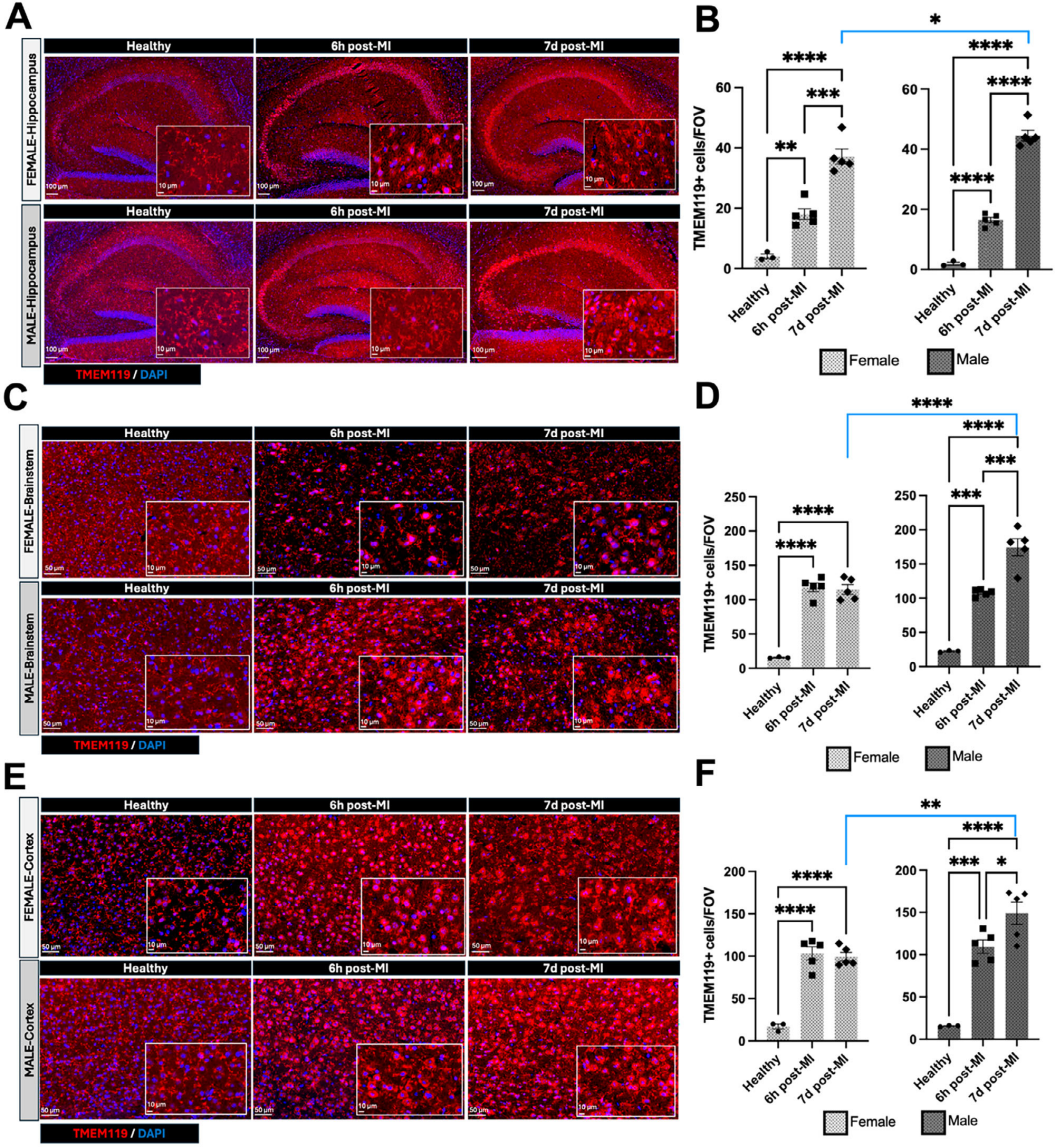
The number of activated microglia increases in the brain post‐MI. (A) Representative immunofluorescence images of TMEM119 in the hippocampus. (B) Quantification of the number of TMEM119^+^ cells per field‐of‐view, scale bar = 100 µm (10 µm for inset). (C) Representative immunofluorescence images of TMEM119 in the brainstem. (D) Quantification of the number of TMEM119^+^ cells per field‐of‐view, scale bar = 50 µm (10 µm for inset). (E) Representative immunofluorescence images of TMEM119 in the cortex. (F) Quantification of the number of TMEM119^+^ cells per field‐of‐view, scale bar = 50 µm (10 µm for inset). Data are presented as mean ± SEM (*n* = 3 for healthy and *n* = 5 for 6 h and 7d post‐MI groups). Differences between groups were determined by a one‐way ANOVA (black line), and differences between sexes were determined by a two‐way ANOVA (blue line). ^*^ *p* < 0.05, ^**^ *p* < 0.01, ^***^ *p* < 0.001, ^****^ *p* < 0.0001.

Having observed increased total NF‐κB and IL‐10 expression in the brain post‐MI, we further investigated the neuroinflammatory response by examining the activation of microglial cells. Microglia are resident immune cells and the first to respond to brain injury or disease [26] by altering their morphology from a resting state to their activated microglia phenotype [27, 28]. To assess microglia activation in the different brain regions after MI, immunohistochemistry was performed on tissue sections using transmembrane protein 119 (TMEM119) [29] as a marker for microglia, and the activated cells were identified by their characteristic round amoeboid morphology. The results demonstrate that the number of activated microglia is increased in all brain regions at 6 h and 7 days post‐MI compared to the healthy group for both females and males (Figure 6A–F; Figure S6A–D). Moreover, the number of activated microglia increased between 6 h and 7 days post‐MI in the hippocampus of female mice and in the hippocampus, brainstem, cortex, and cerebellum of male mice (Figure 6B,D,F; Figure S6D). Notably, at 7 days post‐MI, males exhibited a greater number of activated microglia in the hippocampus, brainstem, cortex, and cerebellum compared to females (Figure 6B,D,F; Figure S6D). These findings identify that microglial cells in the brain are activated as early as 6 h after MI in mice, and that they remain activated for at least 1 week, with greater numbers observed in males compared to females, even increasing in number over this time period in some brain regions.

### 2.5 TNF‐α is Up‐Regulated in the Brain after MI

**FIGURE 7:**
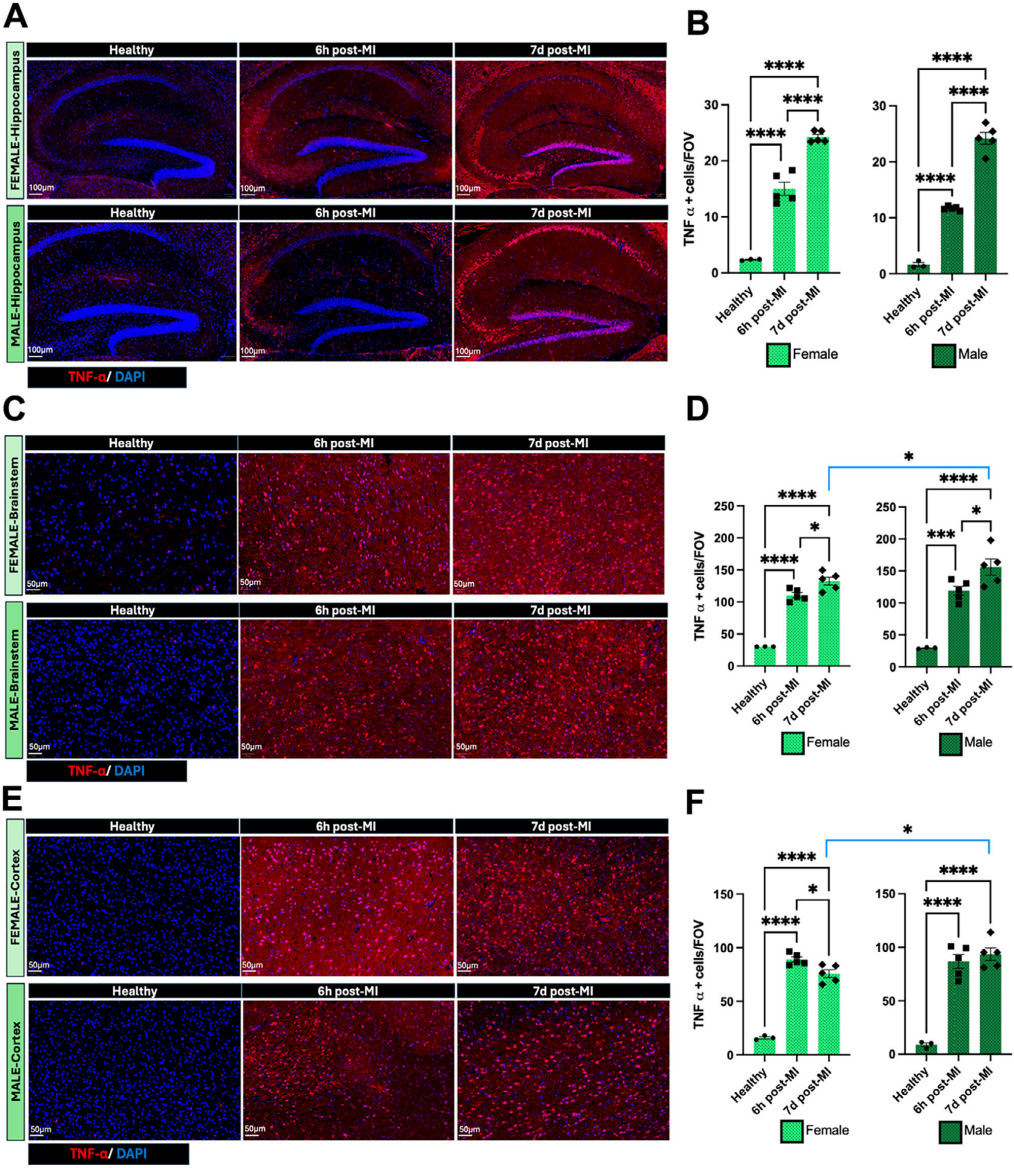
TNF‐α levels increase in the brain post‐MI. (A) Representative immunofluorescence images of TNF‐α in the hippocampus. (B) Quantification of the number of TNF‐α^+^ cells per field‐of‐view, scale bar = 100 µm. (C) Representative immunofluorescence images of TNF‐α in the brainstem. (D) Quantification of the number of TNF‐α^+^ cells per field‐of‐view, scale bar = 50 µm. (E) Representative immunofluorescence images of TNF‐α in the cortex. (F) Quantification of the number of TNF‐α^+^ cells per field‐of‐view, scale bar = 50 µm. Data are presented as mean ± SEM (*n* = 3 for healthy and *n* = 5 for 6 h and 7d post‐MI groups). Differences between groups were determined by a one‐way ANOVA (black line), and differences between sexes were determined by a two‐way ANOVA (blue line). ^*^ *p* < 0.05, ^****^ *p* < 0.0001.

Microglial cells can be activated by tumor necrosis factor‐alpha (TNF‐α), which is a pro‐inflammatory cytokine that plays a key role in the initiation and regulation of the inflammatory response [30]. Also, activated microglia are a main source of TNF‐α, further propagating the inflammatory process [31]. Therefore, we used immunofluorescence staining of TNF‐α to further assess inflammation in the brain post‐MI. Our results show that TNF‐α levels increased in all brain regions at 6 h and/or 7 days post‐MI compared to the healthy group for both female and male mice (Figure 7A–F; Figure S7A–D). Furthermore, there was greater TNF‐α expression at 7 days compared to 6 h post‐MI in the hippocampus for both females and males, and in the brainstem for males. Regarding the sex‐related comparison, male mice were observed to have higher TNF‐α expression than females in the brainstem and cortex at 7 days post‐MI (Figure 7D,E). These results reveal that a greater number of activated microglia in various brain regions after MI is associated with higher production of the inflammatory cytokine TNF‐α, and that these increases are more pronounced in males than females for many brain regions.

### 2.6 The Number of Macrophages is Increased in the Brain Post‐MI

**FIGURE 8:**
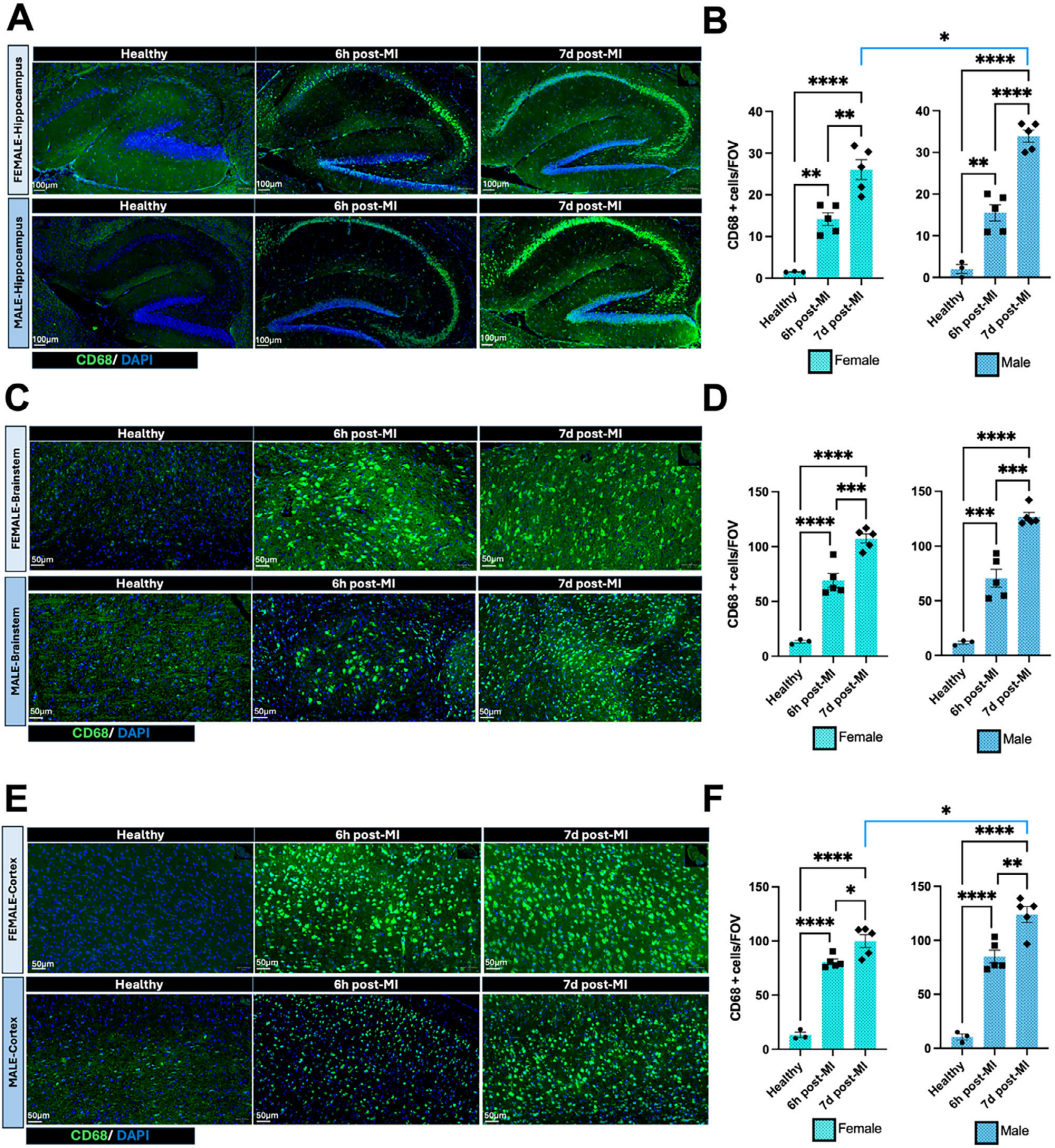
The number of total macrophages increases in the brain post‐MI. (A) Representative immunofluorescence images of CD68^+^ cells in the hippocampus. (B) Quantification of the number of CD68^+^ cells per field‐of‐view, scale bar = 100 µm. (C) Representative immunofluorescence images of CD68^+^ cells in the brainstem. (D) Quantification of the number of CD68^+^ cells per field‐of‐view, scale bar = 50 µm. (E) Representative immunofluorescence images of CD68^+^ cells in the cortex. (F) Quantification of the number of CD68^+^ cells per field‐of‐view, scale bar = 50 µm. Data are presented as mean ± SEM (*n* = 3 for healthy and *n* = 5 for 6 h and 7d post‐MI groups). Differences between groups were determined by a one‐way ANOVA (black line), and differences between sexes were determined by a two‐way ANOVA (blue line). ^*^ *p* < 0.05, ^**^ *p* < 0.01, ^***^ *p* < 0.001, ^****^ *p* < 0.0001.

Finally, to further investigate neuroinflammation, we assessed the total macrophage population in the brain following MI using CD68 as a pan‐macrophage marker [32]. The results demonstrated that the number of CD68^+^ cells increased in all brain regions of female and male mice at 6 h and 7 days post‐MI compared to the healthy group (Figure 8A–F; Figure S8A–D). In addition, the number of CD68^+^ cells increased between 6 h and 7 days post‐MI in the hippocampus and brainstem of females and males (Figure 8B,D), and in the cortex of male mice (Figure 8F). Compared to female mice, males had a greater number of CD68^+^ cells in the hippocampus and cortex at 7 days post‐MI (Figure 8B,F). These findings indicate that macrophages populate the brain after MI, that their numbers increase from 6 h to 7 days, and that males have greater numbers than females in specific brain regions.

### 2.7 Elevated MG‐H1 Levels Correlate with Inflammatory Markers in the Brain Post‐MI

**FIGURE 9:**
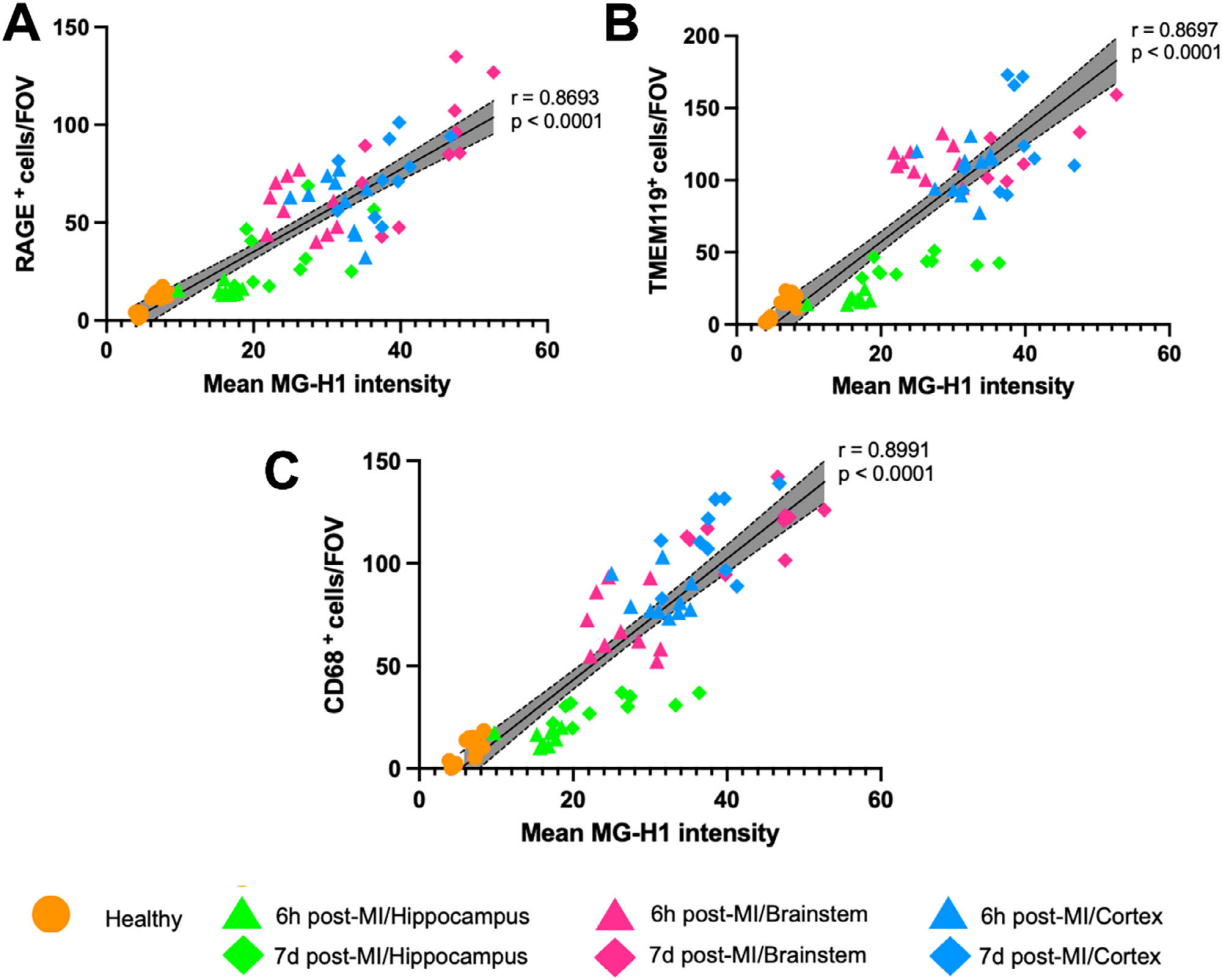
Brain MG‐H1 levels correlate with markers of neuroinflammation post‐MI. (A–C) Correlation analysis of MG‐H1 level vs. RAGE (A), TMEM119 (activated microglia) (B), and CD68 (macrophages) (C). Male and female samples of the hippocampus, brainstem, and cortex regions for the healthy, 6 h post‐MI, and 7d post‐MI groups were used for correlation analysis. The *r* value represents the Pearson correlation coefficient. *R^2^* values for MG‐H1 vs. RAGE, TMEM119, and CD68 were 0.7556, 0.7533, and 0.8084, respectively.

Having observed elevated MG‐H1 and inflammatory markers in the brain post‐MI, a correlation analysis was performed to further assess the association between MG‐H1 levels and neuroinflammatory responses. To this end, we used immunohistochemistry results for MG‐H1 compared to the expression of RAGE, TMEM119, and CD68 markers. Our analysis revealed a strong positive correlation between the level of MG‐H1 and all three markers (Figure 9). These findings suggest that MG‐H1 accumulation in the brain post‐MI is closely associated with both receptor‐mediated glycation stress (RAGE) and the presence of inflammatory cells (activated microglia and macrophages), implicating MG‐H1 as a potential driver of neuroinflammation in the context of cardiac injury. To examine the relationship between scar size and MG‐H1 accumulation and neuroinflammation in the brain, the levels of MG‐H1, RAGE, and TMEM119 at 7 days post‐MI were normalized to scar size for each mouse. While some trends were observed for an increased ratio in males (RAGE and TMEM119 in the brainstem, and MG‐H1 and TMEM119 in the cortex; 0.05 < *p* < 0.15), there were no statistically significant differences between the sexes (Figure S10).

### 2.8 Breakdown of Blood‐Brain Barrier Post‐MI

**FIGURE 10:**
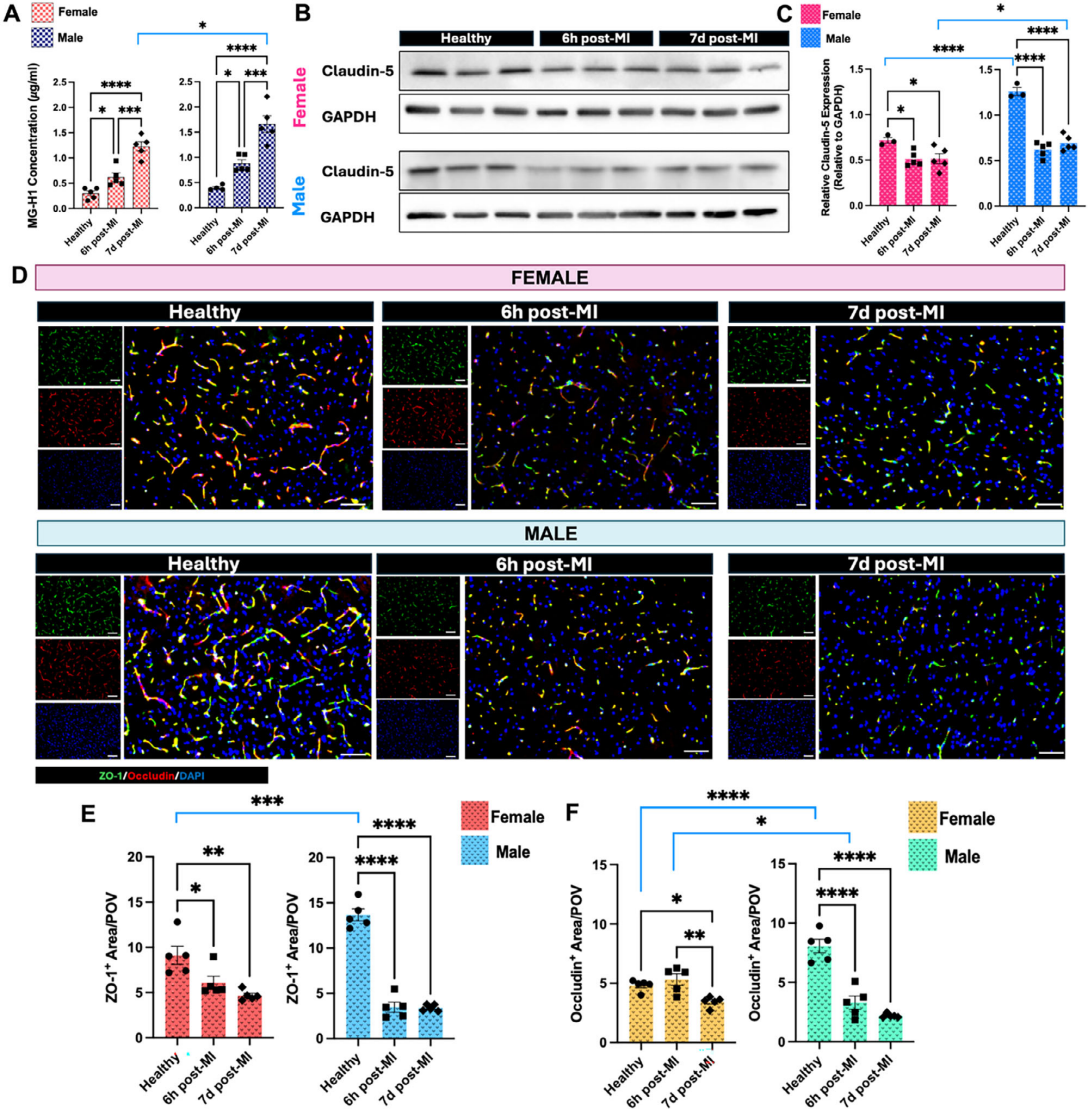
Circulating MG‐H1 levels increase, and brainstem tight junction protein expression decreases post‐MI. (A) Quantification of MG‐H1 in serum of mice by ELISA (*n* = 4–5 per group). (B) Representative western blot images of claudin‐5 in the brainstem. (C) Quantification of claudin‐5 relative to α‐tubulin (*n* = 3 for healthy and *n* = 5 for 6 h and 7d post‐MI groups). (D) Representative immunofluorescence images of ZO‐1 and occludin staining for female and male mice, scale bar = 50 µm. (E,F) Quantification of ZO‐1^+^ (E) and occludin^+^ (F) area per field‐of‐view (*n* = 5 for all groups). Differences between groups were determined by a one‐way ANOVA (black line), and differences between sexes were determined by a two‐way ANOVA (blue line). ^*^ *p* < 0.05, ^**^ *p* < 0.01, ^***^ *p* < 0.001, ^****^ *p* < 0.0001.

As discussed, circulating MG‐AGEs are elevated in patients after MI [14, 15, 16]. In this mouse study, we observed increasing MG‐H1 levels in the serum of both female and male mice from 6 h to 7 days post‐MI compared to baseline, with males having more MG‐H1 in the blood at 7 days compared to females (Figure 10A). Furthermore, the level of circulating MG‐H1 positively correlated with the amount of MG‐H1 that accumulated in the different brain regions at 6 h and 7 days post‐MI (Figure S11). The blood‐brain barrier (BBB) is a structure that serves as a protective mechanism by limiting the free diffusion of substances between the blood and brain [33, 34]. Studies on neurological disorders have identified that MG‐AGEs can cause a loss of BBB integrity, allowing them to cross the BBB [21, 35], and that this involves a reduction in the expression of its tight junction proteins [36, 37, 38, 39]. Therefore, we examined the breakdown of the BBB as a source for the brain entry of MG‐H1 from the circulation post‐MI. To this end, we assessed the expression of the tight junction proteins zonula occludens‐1 (ZO‐1), occludin, and claudin‐5, which are critical to maintaining BBB integrity [33]. Our findings revealed a decrease in the expression of these tight junction proteins in multiple brain areas after MI compared to baseline (Figure 10; Figures S12 and S13). For example, ZO‐1 and claudin‐5 were decreased in the brainstem of females and males at 6 h and 7 days post‐MI, while occludin was decreased only in males at 6 h and in both sexes at 7 days (Figure 10B–F). In males, ZO‐1, occludin, and/or claudin‐5 expression was reduced at 6 h and 7 days post‐MI in all other brain regions except the cerebellum, whereas females had reduced expression only for occludin in the cortex and hippocampus at 7 days post‐MI (Figures S12 and S13). Regarding sex differences, compared to healthy females, healthy males had higher expression of ZO‐1, occludin, and claudin‐5 in the brainstem, ZO‐1 in the cortex and hippocampus, and occludin in the rest of the brain (Figures S12 and S13). However, after MI this pattern was lost, with female mice exhibiting greater expression of tight junction proteins in some brain regions (occludin in the brainstem at 6 h post‐MI, and ZO‐1 and occludin in the cortex and cerebellum at 6 h post‐MI).

## 3 Discussion

The importance of the bidirectional crosstalk between the heart and the brain is increasingly being recognized as a critical factor in the development and progression of cardiovascular and neurodegenerative diseases. However, there is still much to understand about how cardiovascular diseases affect the brain and how neurodegenerative diseases can impact heart health. In this study, we investigated a possible role for MG in the heart‐brain axis and report for the first time that MG‐AGEs accumulate in the brain post‐MI, that this is correlated with increased neuroinflammation, and that these effects vary in different brain regions and between the sexes. Our findings identify a novel mechanism with sex‐based differences that may contribute significantly to heart‐brain interactions after MI and the neurological impairment often associated with heart disease.

Clinical studies have shown that patients with MI have a higher incidence of behavioral disorders such as depression, and that both MI and heart failure are associated with an increased risk of developing neurological disorders (e.g., dementia, including Alzheimer's disease) and cognitive impairment [3, 4, 5, 6, 40]. In addition, preclinical studies have shown that MI causes depression‐like behavior [7] and cognitive decline [41, 42] in animal models. One study showed that male rats developed depression‐like behavior at 10 weeks post‐MI [43]. Despite these strong links between the health of the heart and the brain, the underlying cause or mechanism of the depression‐like behavior or cognitive decline remains to be better elucidated.

MG is a promising candidate to consider for a role in heart‐brain interactions in disease, as it is a shared causative factor associated with several pathological conditions, such as diabetes [13, 44], heart disease [10, 45], and neurodegenerative disease [46, 47]. Much of the damage caused by MG in these and other diseases is through the generation of oxidative stress and inflammation [48]. Levels of this highly reactive dicarbonyl compound are increased acutely in the myocardium after MI, leading to the accumulation of MG‐AGEs in the heart and other tissues [10, 14, 18]. Given this evidence and the fact that MG is a risk factor for developing neurological disorders and cognitive impairment [49], we hypothesized that MI leads to MG‐AGE accumulation and neuroinflammation in the brain.

Our results demonstrated that the level of MG‐H1 in the five different brain regions was increased after MI compared to the healthy controls. It was also observed that MG‐AGE accumulation varied between the different brain regions, with the brainstem exhibiting the highest MG‐H1 expression in both females and males at 7 days post‐MI. Notably, a greater level of MG‐H1 was observed in the brainstem, cortex, and hippocampus of male mice compared to females at 6 h and/or 7 days post‐MI. Sex‐related differences in heart‐brain interactions have been reported to affect the development of heart and brain diseases [50, 51, 52], but little is known about the underlying mechanism(s) involved. Our data suggest that MG‐AGEs may be a factor contributing to sex‐based differences in brain damage and neurological disorders that develop after MI. Males sustain greater damage and develop larger scars after MI compared to females, which has been attributed in part to the protective effect of estrogen in females and an exacerbation effect of testosterone in males [53, 54, 55, 56]. This suggests that the increased MG‐H1 and neuroinflammation observed in male brains in our study may be related to greater myocardial damage post‐MI. When brain levels of MG‐H1, RAGE, and TMEM119 were normalized to scar size, there were a few trends for increased susceptibility of males to MG‐mediated damage in some brain regions compared to females, but no statistically significant differences. Thus, smaller infarctions in females are likely contributing to the reduced levels of MG‐H1 and neuroinflammation observed in their brains at 1‐week post‐MI. This is further supported by the observation that circulating levels of MG‐H1 post‐MI were lower in females than in males. Taken together, our results revealed that MG‐AGEs accumulate in the brain post‐MI and that this varies between brain regions and between the sexes.

Glo1 is a key enzyme that metabolizes MG in normal conditions, but conditions such as inflammation and oxidative stress lead to increased MG production while also limiting Glo1 activity [13]. Interestingly, Glo1 expression was significantly increased after MI only in the brainstem and cortex of male mice, which coincided with the regions expressing the greatest level of MG‐AGEs. Despite the greater presence of Glo1 in these regions, the level of MG‐AGEs increased in the tissue over time, suggesting that the defence mechanism was incapable of keeping up, possibly through reduced Glo1 activity [13]. In contrast, Glo1 expression did not change after MI in the brains of female mice. Although female brains also exhibited increased MG‐AGEs, this was consistently lower than the levels observed in males, suggesting that the Glo1 activity in females may be able to limit some of the damaging effects of MG. Given these observed differences, further evaluation of the glyoxalase system post‐MI would constitute a rational next step for future research, including an assessment of Glo1 activity and levels of its co‐factor reduced glutathione. Taken together, like MG‐H1, Glo1 expression varies in different brain regions and between the sexes after MI.

It is known that MG levels in the circulation are increased after MI [14, 15, 16], and studies have shown that MG can cause a loss of blood‐brain barrier (BBB) integrity and can cross the BBB [21, 35]. MG‐mediated breakdown of the BBB involves a reduction in the expression of critical tight junction proteins [36, 37, 38, 39]. Our results showed a significant decrease in the expression of the tight junction proteins ZO‐1, occludin, and claudin‐5 in most brain regions post‐MI, with the greatest reduction observed in the brainstem in both females and males. This loss of BBB integrity would provide a means for MG‐H1 to enter the brain from the circulation. Notably, increased levels of circulating MG‐H1 were correlated with greater levels of MG‐H1 in the brain. Together, these results support that MG‐H1 from the circulation crossing the BBB is a probable source for its accumulation in the brain post‐MI. Inflammatory cytokine levels are also increased in the circulation after being released from the heart post‐MI and have been shown to disrupt the BBB [7, 57], potentially providing another means for the entry of MG and its AGEs into the brain. Another possibility is that cytokine signaling post‐MI may cause inflammation in the brain, which could subsequently increase MG levels since inflammation can promote MG production [58, 59]. As both MG and inflammatory cytokines are elevated in the blood post‐MI, it is likely that these mechanisms work synergistically to increase MG, oxidative stress, and inflammation in the brain, which constitutes an area for future investigation.

Since MG and its AGEs induce inflammation, we assessed if the increased presence of MG‐AGEs in the brain post‐MI was associated with greater neuroinflammation. Studies have shown that MG exposure triggers increased expression of RAGE [60, 61], and that RAGE signaling leads to the production of ROS, inflammation, oxidative stress, and pro‐inflammatory cytokines [11]. The immunofluorescence staining results revealed a significant increase in RAGE expression post‐MI in all brain regions of both female and male mice compared to healthy controls. Additionally, males exhibiting higher RAGE levels in the hippocampus, brainstem, and cortex compared to females at 6 h and/or 7 days post‐MI. Notably, these results aligned with the observed MG‐H1 expression patterns.

The observed increase in RAGE expression in the brain post‐MI was accompanied by the presence of neuroinflammation in the various brain regions, as evidenced by a greater number of activated microglia and total macrophages, as well as increased levels of the transcription factor NF‐κB and cytokines involved in regulating inflammation (IL‐10, TNF‐α) in female and/or male mice. Although there are some reports on how MI affects these markers of neuroinflammation for some brain regions, to our knowledge, our study is the first to assess these changes temporally in all brain regions, to identify sex‐based differences, and to associate the inflammation with MG accumulation. Microglial activation is a key mediator of neuroinflammation through the promotion pro‐inflammatory factor expression [26, 62]. Previous studies have reported that the number of microglia increases significantly in the cortex of mice and rats at up to 7 days post‐MI compared to sham controls [63, 64]. Similarly, we observed a greater number of activated microglia in both female and male mice at 6 h and 7 days post‐MI in the cortex and in all the other brain regions, compared to healthy animals, with the brainstem and cortex exhibiting greater microglial activation at both time points. Notably, in the hippocampus, brainstem, cortex and cerebellum, a significant increase in activated microglia was observed at 7 days compared to 6 h post‐MI in males, while in females, this temporal increase was restricted to the hippocampus. Also, males had significantly greater microglial activation at 7 days post‐MI in the hippocampus, brainstem, cortex and cerebellum, relative to females. It is known that MG can shift microglial cells toward a pro‐inflammatory phenotype [21]. In vivo studies have shown that a reduction of MG and MG‐AGEs in microglia reduces the number of activated microglia [65]. Since males exhibited higher levels of MG‐AGEs than females in the present study, this may explain their greater number of activated microglia.

Microglia are a primary source for TNF‐α, a pro‐inflammatory cytokine that plays an important role in the neuroinflammatory response associated with neurological disease, including increased BBB permeability [7, 31, 66]. Concerning heart‐brain interactions, clinical studies have reported that patients with heart failure and major depressive disorder have high plasma levels of TNF‐α [67, 68]. Furthermore, Najjar et al. [43] showed that pro‐inflammatory cytokines such as TNF‐α are increased in the cortex of rats after MI. In the present study, the number of TNF‐α^+^ cells were increased in all brain regions at 6 h and/or 7 days post‐MI in females and males compared to healthy mice, with the highest numbers observed in the cortex and brainstem for each sex group. Furthermore, males had a greater number of TNF‐α^+^ cells than females in the brainstem and cortex at 7 days post‐MI. Microglia can also polarize toward an M2 anti‐inflammatory phenotype, which is accompanied by increased production of IL‐10 [69]. The upregulation of IL‐10 observed in some brains regions in mice post‐MI suggests activation of compensatory anti‐inflammatory signaling, possibly by the presence of repair‐associated microglial cells.

The increased presence of macrophages is another sign of inflammation. Thackeray et al. [64] identified that the number of CD68^+^ cells is increased in the brain at 1‐week post‐MI in mice. In this study, CD68 immunofluorescence staining was done to assess the number of macrophages in different brain regions post‐MI. Since CD68 is a pan‐macrophage marker, its use identified both recruited macrophages and resident macrophages (i.e., activated microglia in the brain that express CD68 [70]). Results showed an increase in the number of CD68^+^ cells in all brain regions of female and male mice after MI. Similar to the other markers that were assessed, the number of CD68^+^ cells was higher in the brainstem and cortex compared to the other brain regions. Sex differences in CD68^+^ cell numbers were also observed, with males having higher numbers in the hippocampus, cortex and the rest of the brain, compared to females.

Overall, our findings suggest that elevated levels of MG and its AGEs in the brain post‐MI may promote microglial and/or macrophage activation as a mediator of neuroinflammation. Notably, the brainstem exhibited the highest MG‐H1 level among all regions analyzed, as well as the greatest increase of RAGE expression and presence of neuroinflammatory markers (activated microglia, macrophages, and inflammatory cytokines). The cortex had the second‐highest expression of MG‐H1, RAGE, and inflammatory markers after MI, which was less than in the brainstem, but greater than the other 3 brain regions analyzed. This highlights a region‐specific vulnerability to glycation‐induced stress post‐MI. Interestingly, a brain‐wide mapping study was recently reported, looking at brain regions associated with the input and output of the heart, and it identified areas within the brainstem and cortex as being key regulators of brain‐heart interconnectivity [71]. Given this, our findings suggest that the prevalent MG‐AGE accumulation and neuroinflammation in the brainstem and cortex likely play a role in the dysregulation of important brain‐heart interactions after MI.

MG‐AGEs play an important role in the development of neurodegenerative disorders such as dementia, including Alzheimer's disease [72, 73], and in the pathogenesis of mental health challenges (including depression) and cognitive impairment (such as memory loss) [21, 74, 75, 76]. Given the observation that MG‐AGE levels were increased acutely in the brains of mice post‐MI, the present study strongly suggests that MG may be an underlying cause for the increased prevalence of these brain disorders in patients with MI. However, a limitation of this study is that the long‐term expression of the MG‐AGEs and their effects on the brain and mouse behavior were not assessed. As such, the association we propose linking MG‐AGE accumulation in the brain post‐MI to the increased prevalence of neuropsychiatric disorders is based on evidence from prior human studies, rather than demonstrated experimentally in this model. This would constitute an important direction for future investigation. Another consideration is that our study's control group consisted of healthy mice, and a SHAM group was not included. We wanted to make a comparison between the brain post‐MI and a healthy brain without any activated systemic inflammation. As such, a possible contribution of systemic inflammation due the surgical procedure itself (rather than the MI) to the observed effects on the brain cannot be ruled out. However, given that the level of MG‐H1 and neuroinflammation in the brain post‐MI was related to the severity of MI and to the circulating levels of MG‐H1, this highly supports that the MI is primarily responsible for the effects on the brain.

## 4 Conclusion

In summary, to our knowledge, this is the first study reporting on the accumulation of MG‐AGEs in the brain post‐MI. MG‐H1 accumulation was greatest in the brainstem and cortex and was correlated with increased neuroinflammation. Notably, there were temporal, regional, and sex‐based differences in the observed MG‐H1 expression and neuroinflammation, thus identifying distinct neuroimmune responses to cardiac injury. This highlights a novel mechanism of MG accumulation and neuroinflammation that may play an important role in heart‐brain interactions and the pathophysiology of neurodegenerative disorders after MI. It also identifies a promising therapeutic target to consider for mitigating neurological impairment associated with heart disease.

## 5 Experimental Section

### 5.1 Myocardial Infarction Model

All animal procedures were approved by the University of Ottawa Animal Care Committee (protocol # HIe‐4260; approved on 3/21/2025) and performed according to the National Institute of Health Guide for the Care and Use of Laboratory Animals. MI was induced by permanent left coronary artery ligation surgery in 8‐week‐old male and female C57BL/6 mice (Charles River), as previously reported [10, 45]. Briefly, mice were anesthetized (2% isoflurane), intubated, and the heart was exposed via a fourth intercostal thoracotomy. The left anterior descending coronary artery was then ligated just below its emergence from the left atrium. This procedure resulted in a mid/large MI involving the anterolateral, posterior, and apical parts of the left ventricle, which was confirmed at the time of surgery by myocardial blanching in the region supplied by the artery. Short‐acting buprenorphine was administered at least an hour prior to surgery, and long‐acting meloxicam was administered subcutaneously immediately before surgery for perioperative analgesia. See the experimental timeline provided in Figure 1A.

### 5.2 Tissue Collection for Molecular Analysis

Brain and heart tissues were collected at 6 h and 7 days post‐MI, and control brain and heart tissue were collected from age‐matched non‐infarcted mice (healthy). After washing with ice‐cold PBS, the whole brain tissues were then freshly dissected into five different brain regions, namely the hippocampus, cerebral cortex, cerebellum, brainstem, and the rest of the brain (see the dissected brain regions schematic in Figure 1B). Dissected brain tissues and hearts were immediately snap frozen in liquid nitrogen and stored at −80°C until further use.

### 5.3 Echocardiography

Cardiac function was analyzed by echocardiography at baseline prior to MI surgery and at 6 h and 7 days post‐MI. Using a Vevo3100 system with an MX400 series real‐time microvisualization scan head probe (VisualSonics), transthoracic echocardiography was performed in B mode to capture the long‐axis view of the left ventricle. Vevo LAB 3.1.1 software (VisualSonics) was used to calculate left ventricular ejection fraction (LVEF).

### 5.4 ELISA Analysis

To quantify MG‐H1 levels in the serum, an ELISA kit (Cell Biolabs, Cat# STA‐811) was used. Blood was collected and left undisturbed at room temperature for 30 min to allow clotting. The clot was then removed by centrifuging at 2000 g for 15 min at 4°C, and the supernatant (serum) was transferred to new tubes. The serum samples were analyzed using the ELISA kit according to the manufacturer's protocol. The absorbance of samples was measured using a microplate reader (Agilent BioTek Synergy H1 Hybrid Multi‐Mode Reader) at 450 nm. The concentrations of samples were determined by comparison with a standard curve generated from known standards.

### 5.5 Western Blot Analysis

Brain tissues were lysed with ice‐cold RIPA buffer containing protease/phosphatase inhibitor cocktail (Roche, Cat# 04693159001), and supernatants were centrifuged at 12 000 g for 30 min at 4°C. The total protein concentrations were then measured using a Pierce BCA Protein Assay Kit (Fisher Scientific, Cat#PI23225) according to the manufacturer's protocol. Samples were heated at 95°C for 5 min, and equal amounts of sample (30 µg) were loaded to a 4%–10% or 4%–12% SDS gel and run at 100 V for 90 min in running buffer. Proteins were transferred to PVDF membranes (Bio‐Rad Laboratories) at 100 V for 90 min in transfer buffer. Membranes were then blocked for 1 h at room temperature with 3% or 5% skim milk in Tris‐buffered saline‐Tween20 (TBS‐T). The immunoblots were probed with anti‐MG derived AGE antibody (Novus Biologicals, Cat#NBP2‐62810 at 1:1000 dilution), anti‐Glo1 (Novus Biologicals, Cat#NBP1‐31466 at 1:1000 dilution), anti‐NF‐κB p65 (Santa Cruz, Cat#sc‐8008 at 1:1000 dilution), anti‐IL‐10 (Abcam, Cat#ab9969 at 1:1000 dilution), anti‐claudin 5 (Invitrogen, Cat#34‐1600 at 1:500), anti‐GAPDH (Santa Cruz, Cat#sc‐59540 at 1:1000 dilution) and anti‐α‐tubulin (Cell Signaling, Rabbit, Cat#2125 at 1:1000 dilution) primary antibodies overnight. After washing with TBS‐T several times, membranes were incubated with anti‐rabbit (Novus Biologicals, HRP, Cat#N7185 at 1:10000 dilution) or anti‐mouse (Abcam, HRP, Cat#ab205719 at 1:10000 dilution) secondary antibodies for 1 h at room temperature. For re‐probing, some membranes were washed with TBS‐T and stripped with a stripping buffer and re‐blocked and incubated with other primary antibodies (for NF‐κB p65, IL‐10, and/or α‐tubulin). Protein bands were visualized using the Chemidoc Imaging System. Bands were quantified using densitometry analysis in ImageJ2 software, and intensities of the bands were normalized to α‐tubulin. Briefly, a rectangular box was drawn to the selected protein bands of interest to analyze band densitometry, and the same‐sized rectangular boxes were used to measure all bands.

### 5.6 Immunohistochemistry

Brains were harvested at 6 h and 7 days post‐MI, washed with ice‐cold PBS, and fixed with 10% formalin (Fisher Scientific, cat # SF98‐4). Then, brain tissues were processed with a Spin Tissue Processor (Microm STP‐12, Thermo Scientific) overnight. After the tissue processing step, the tissues were embedded in paraffin for later use. The paraffin‐embedded brain tissues were sectioned (5 µm) using a Lecia Microtome, and sections were mounted onto slides and then dried overnight. The sections were deparaffinized using Leica Autostainer XL (ST5010) prior to the antibody staining protocol. Heart sections were stained using the Masson's trichrome protocol to assess the fibrosis area. For the antigen retrieval step, two different antigen retrieval methods are applied according to the antibody used. For the anti‐TMEM119 antibody, antigen retrieval was performed in a Decloaking Chamber pressure cooker (BioCare Medical) with a citrate buffer (pH 6.0), while for others, the antigen was retrieved in citrate buffer (pH 6.0) for 2 min 50 s in the microwave. The sections were blocked with 10% normal goat serum (BioLynx, Cat#VECTS1000) for 30 min at room temperature and then incubated with primary antibodies overnight at 4°C. For methylglyoxal‐derived hydroimidazolone‐1 (MG‐H1) co‐localization, the protocol was modified in the following ways: triton permeabilization was used, blocking was performed for 2 h at room temperature using anti‐mouse FAB fragment antibody (Jackson Immuno Research 715‐007‐003, 1:25) in 10% normal goat serum. Primary antibodies used for immunostaining were anti‐TMEM119 (1:50, Abcam, Cat#ab209064), anti‐TNF‐α (1:40, Abcam, Cat#ab4183218) and anti‐CD68 (1:500, Abcam, Cat#ab125212), anti‐MG‐H1 (Cell Biolabs cat# STA‐011, 1:100), and anti‐RAGE (1:200, Abcam, cat#ab30381). After primary antibody incubation, sections were washed with 0.05% TBS‐T several times, and then slides were incubated with secondary antibodies at room temperature for 1 h. Secondary antibodies used for immunostaining were AF488 Goat anti‐rabbit secondary (ThermoFisher, Cat#A11008 at 1:500 dilution), AF568 Goat anti‐rabbit secondary (ThermoFisher, Cat#A11036 at 1:500 dilution), and AF488 Donkey anti‐mouse secondary (ThermoFisher, Cat#A21202 at 1:500 dilution). Nuclei were stained with DAPI (1:1000) and then washed several times and mounted with fluorescence mounting medium (Dako, Cat# S3023).

To detect tight junction proteins (ZO‐1 and occludin), antigen retrieval was performed in a Decloaking Chamber pressure cooker (BioCare Medical) using a Tris‐EDTA Buffer pH 9.0 (Abcam, Cat# ab93684). The sections were then permeabilized with 0.1% Triton‐100 for 10 min at room temperature. Next, sections were blocked with 10% normal donkey serum (Abcam, Cat# 7475) containing anti‐mouse FAB fragment antibody (Jackson ImmunoResearch 715‐007‐003, 1:25) for 1 h at room temperature. Primary antibodies used for immunostaining were anti‐ZO‐1 (1:50, Invitrogen, Cat# 33–910) and anti‐occludin (1:50, Abcam, Cat# ab216327). After primary antibody incubation, sections were washed with 0.05% PBS‐T several times. Then, slides were incubated with secondary antibodies at room temperature for 2 h. The secondary antibodies used were AF594 Donkey anti‐Mouse (ThermoFisher, Cat# A21203 at 1:500 dilution) and AF647 Donkey anti‐Rabbit (ThermoFisher, Cat# A31573 at 1:500 dilution). Finally, nuclei were stained with DAPI (1:1000) and washed several times before being mounted with fluorescence mounting medium (Dako, Cat# S3023).

### 5.7 Histological Image Analysis

Immunohistochemistry images were analyzed using QuPath and ImageJ software. To detect positively stained cells, the positive cell detection method was applied for each marker separately (RAGE, TMEM119, TNF‐α, CD68). For each marker, the setup parameters (e.g., threshold and pixel size) were optimized separately and then used to quantify the positively stained cells (Figure S9). For MG‐H1 staining, the mean fluorescent intensity on each selected field‐of‐view was calculated by ImageJ. For ZO‐1 and occludin staining, the mean fluorescent area on each selected field‐of‐view was calculated using ImageJ. At least 3 random images were obtained for each brain region to quantify the number of positive cells/areas/intensities in each field‐of‐view.

### 5.8 Statistical Analysis

Statistical analysis was performed using GraphPad Prism 10.0 software. All data are presented as the mean ± SEM. When comparing more than 2 groups, a one‐way or two‐way analysis of variance (ANOVA) was used, followed by the post hoc Tukey's test. For the correlation analysis, Pearson correlation coefficients were calculated using GraphPad Prism 10.0 to evaluate the relationship between MG‐H1 and the markers RAGE, TMEM119, and CD68. For this, immunohistochemistry data for MG‐H1, RAGE, TMEM119, and CD68 for female and male hippocampus, brainstem, and cortex regions were used, and the data were grouped together by experimental time point (healthy, 6 h and 7 days).

## Author Contributions

E.J.S. and R.I. designed the study. R.I. and X.G. conducted experiments. R.I., X.G., and E.J.S. analyzed the data and/or provided critical insights. E.J.S. provided supervision. R.I. and E.J.S. wrote the manuscript with input from all authors. All authors have reviewed and approved the final version of the manuscript.

## Conflicts of Interest

The authors declare no conflicts of interest.

## Supporting information




**Supporting File**: advs75214‐sup‐0001‐SuppMat.docx.

## Data Availability

All data generated or analyzed in this study are included in the manuscript and the Supporting Information. Other raw and processed data that support the findings of this study are available upon request.
