# Supplementary material for: Methylglyoxal Accumulation is Associated with Brain Inflammation after Myocardial Infarction with Sex and Regional Differences
Source: Adv Sci (Weinh). 2026 Apr 9:e22584. Online ahead of print. doi: 10.1002/advs.202522584 (PMC13334616; doi:10.1002/advs.202522584)
Supplement: Supplementary file 1 — Supporting File: advs75214‐sup‐0001‐SuppMat.docx. [file ADVS-9999-e22584-s001.docx]

**Supporting Information**

Methylglyoxal Accumulation is Associated with Brain Inflammation after Myocardial Infarction with Sex and Regional Differences

**Ramis Ileri, Xixi Guo, and Erik J. Suuronen**

R. Ileri, X. Guo, E. J. Suuronen

Bioengineering and Therapeutic Solutions (BEaTS) program, University of Ottawa Heart Institute, Ottawa, Canada

R. Ileri, E. J. Suuronen

Ottawa-Carleton Institute for Biomedical Engineering (OCIBME), Ottawa, Canada

X. Guo, E. J. Suuronen

Department of Cellular and Molecular Medicine, University of Ottawa, Ottawa, Canada

**
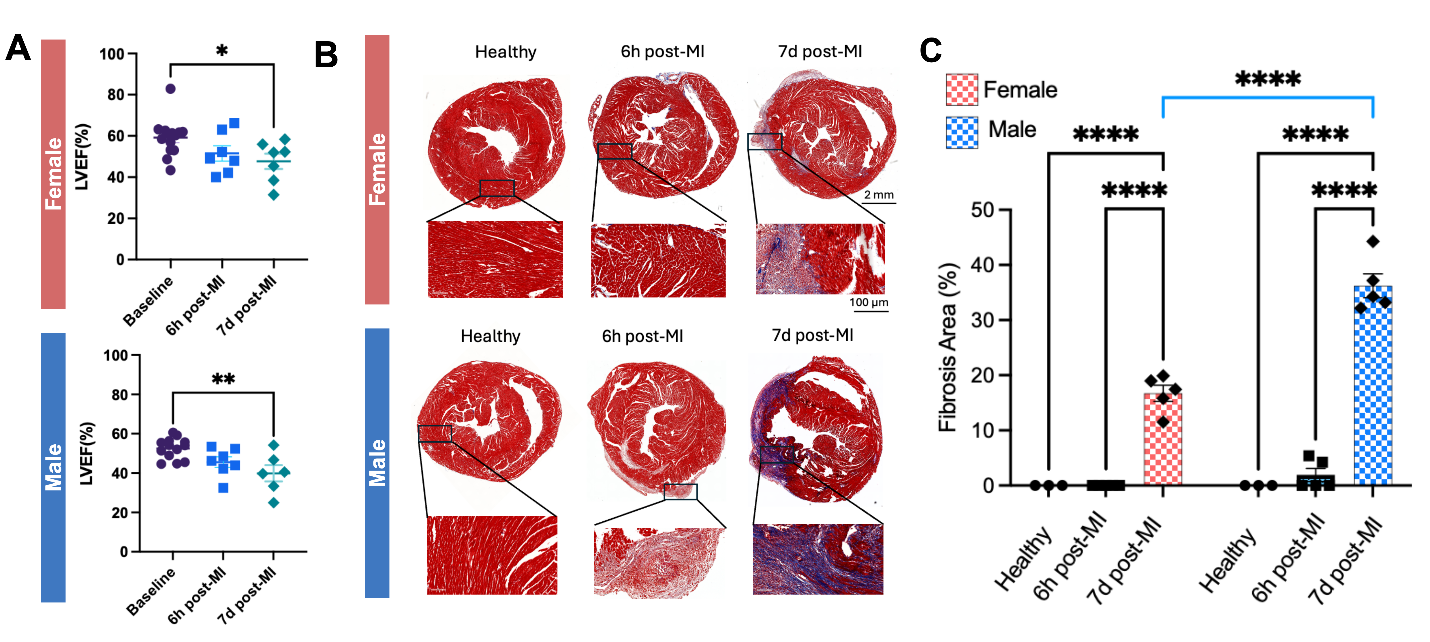
**

**Figure S1.** MI reduces cardiac function and promotes fibrosis. A) LVEF of female and male mice (*n* = 7-14). B) Representative images of Masson’s Trichrome stained sections (*n* = 3 for healthy and *n* = 5 for 6h and 7d post-MI groups). C) Scar size (fibrosis area) was calculated as the % of blue collagen area to total myocardial area. Data are presented as mean ± SEM. Differences between groups were determined by a one-way ANOVA and differences between sexes were determined by a two-way ANOVA (blue line). **p* < 0.05, ***p* < 0.01, *****p* < 0.0001. Created using [BioRender.com](https://www.biorender.com/).


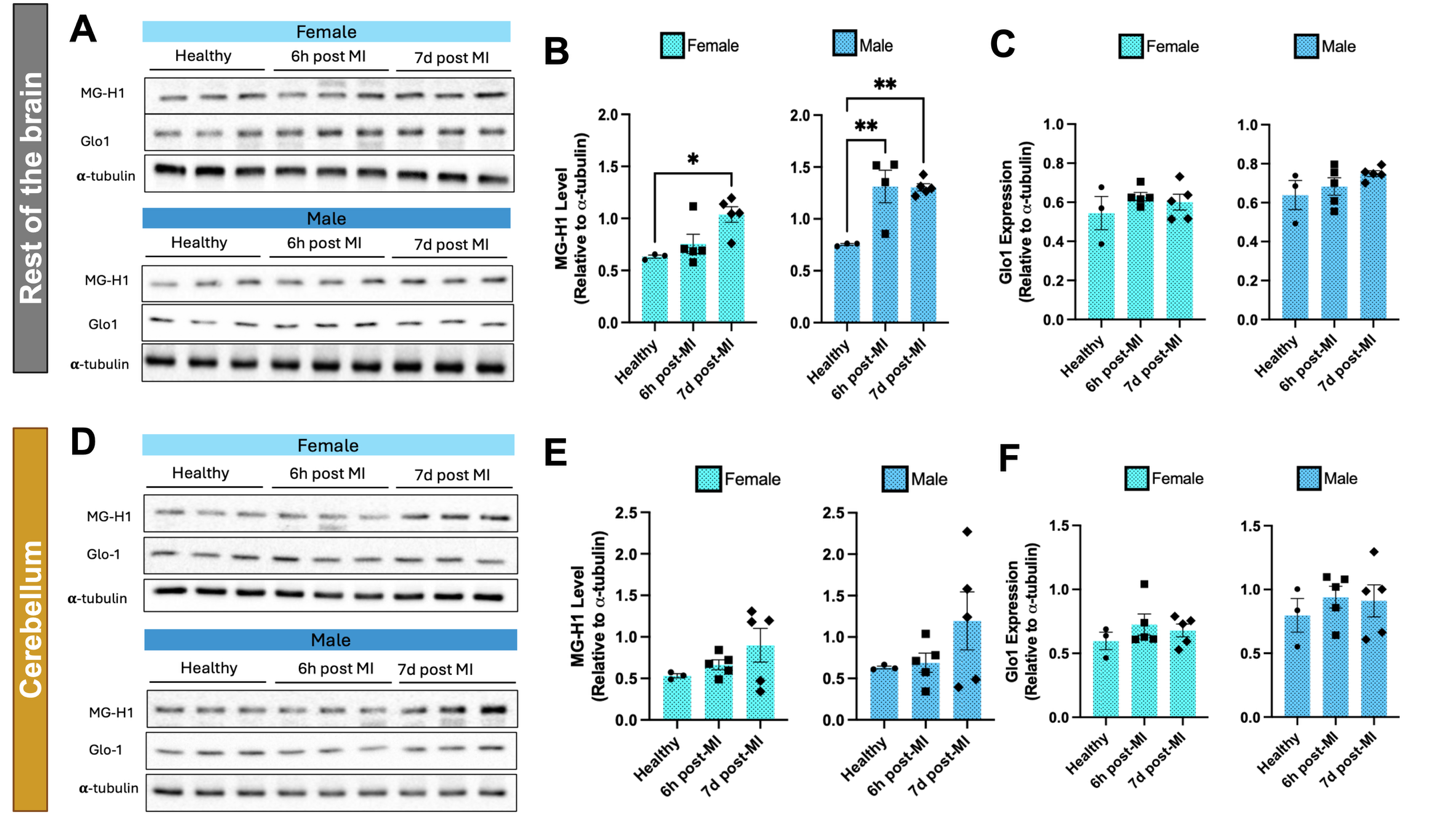


**Figure S2.** MG-H1 increases in the rest of the brain of male mice at 7 days post-MI. A) Representative western blot images for rest of the brain. B, C) Quantification of MG-H1 (B) and Glo1 (C) relative to α-tubulin (*n* = 3 for healthy and *n* = 5 for 6h and 7d post-MI groups). D) Representative western blot images for the cerebellum. E, F) Quantification of MG-H1 (E) and Glo1 (F) relative to α-tubulin (*n* = 3 for healthy and *n* = 5 for 6h and 7d post-MI groups). Data are presented as mean ± SEM. Differences between groups were determined by a one-way ANOVA (black line) and differences between sexes were determined by a two-way ANOVA (blue line). **p* < 0.05, ***p* < 0.01.


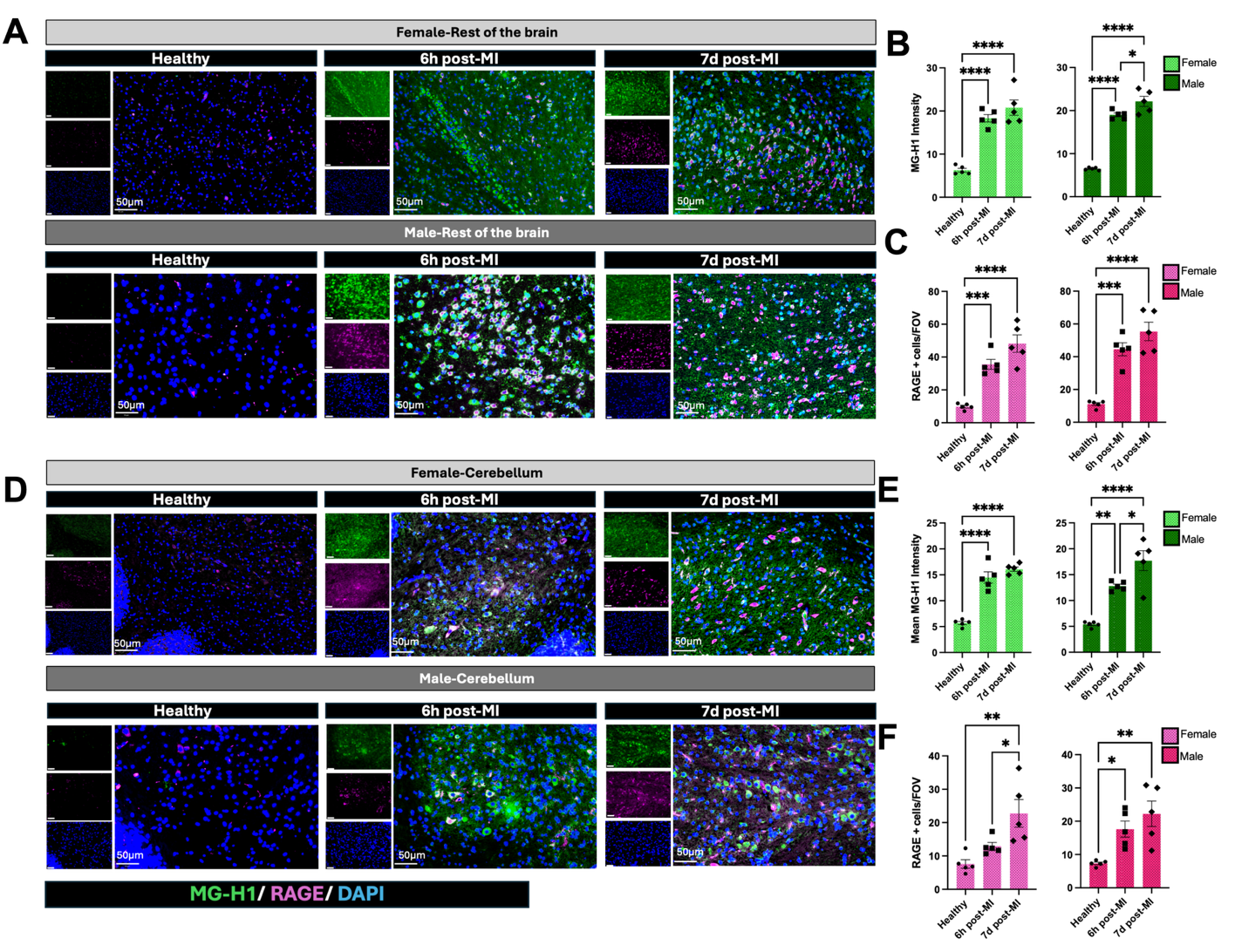


**Figure S3.** MG-H1 and RAGE expression increases in rest of the brain and cerebellum post-MI. A) Representative immunofluorescence images of MG-H1 and RAGE staining in the rest of the brain, scale bar = 50 μm. B, C) Quantification of mean MG-H1 fluorescence intensity (B) and number of RAGE^+^ cells (C) per field-of-view. D) Representative immunofluorescence images of MG-H1 and RAGE staining in the cerebellum, scale bar = 50 μm. E, F) Quantification of mean MG-H1 fluorescence intensity (E) and number of RAGE^+^ cells (F) per field-of-view. Data are presented as mean ± SEM (*n* = 5 per group). Differences between groups were determined by a one-way ANOVA (black line) and differences between sexes were determined by a two-way ANOVA (blue line). **p* < 0.05, ***p* < 0.01, ****p* < 0.001, *****p* < 0.0001.


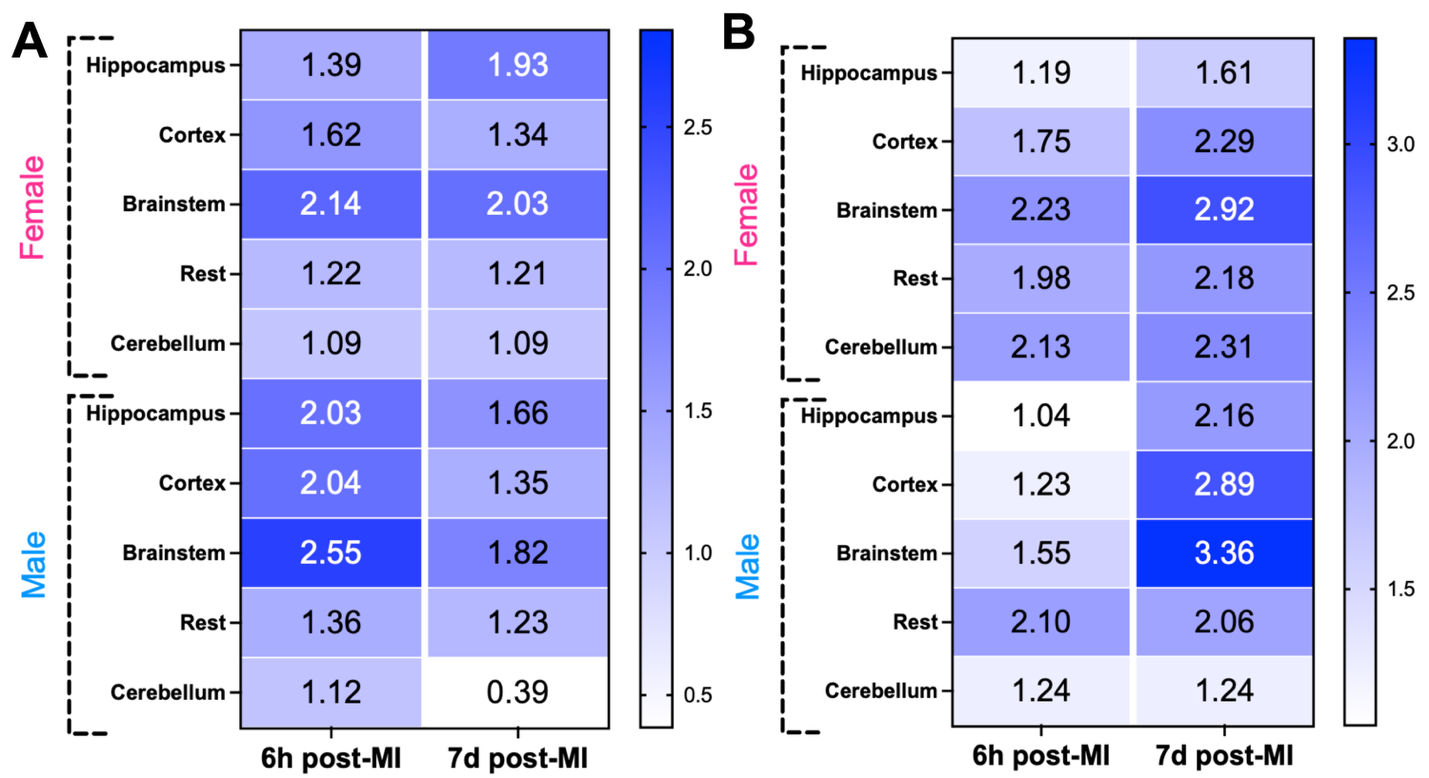


**Figure S4.** Summary of fold changes in total Nf-κB and IL-10 expression. A, B) Fold changes in total NF-κB (A) and IL-10 (B) expression in the brain at 6h and 7d post-MI relative to the healthy group.


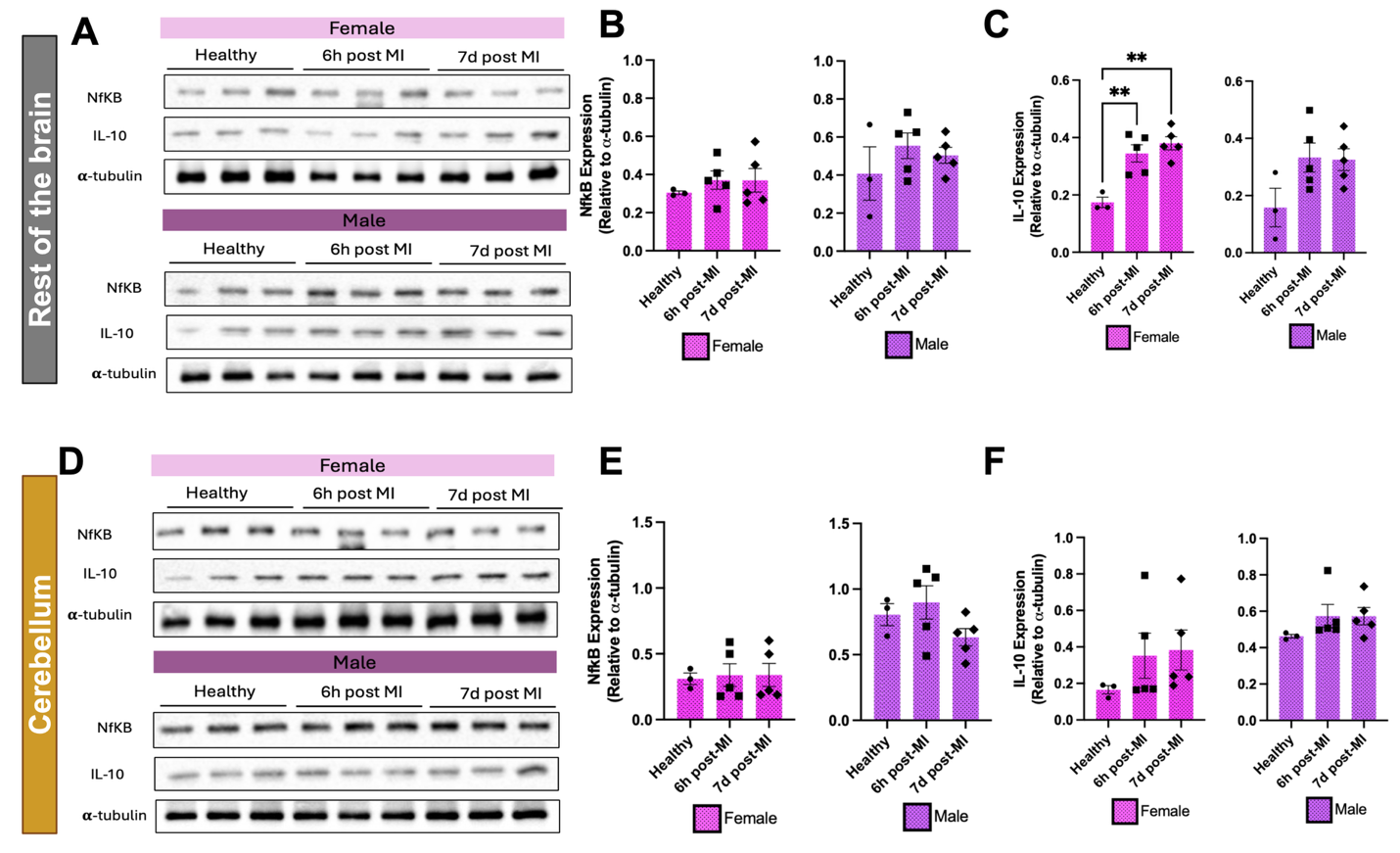


**Figure S5.** Total NF-κB and IL-10 expression in rest of the brain and cerebellum post-MI. A) Representative western blot images for total NF-κB and IL-10 in the rest of the brain. B, C) Quantification of total NF-κB (B) and IL-10 (C) relative to α-tubulin (*n* = 3 for healthy and *n* = 5 for 6h and 7d post-MI groups). D) Representative western blot images for total NF-κB and IL-10 in the cerebellum. E, F) Quantification of total NF-κB (E) and IL-10 (F) relative to α-tubulin (*n* = 3 for healthy and *n* = 5 for 6h and 7d post-MI groups). Data are presented as mean ± SEM. Differences between groups were determined by a one-way ANOVA (black line) and differences between sexes were determined by a two-way ANOVA (blue line). **p* < 0.05, ****p* < 0.001.


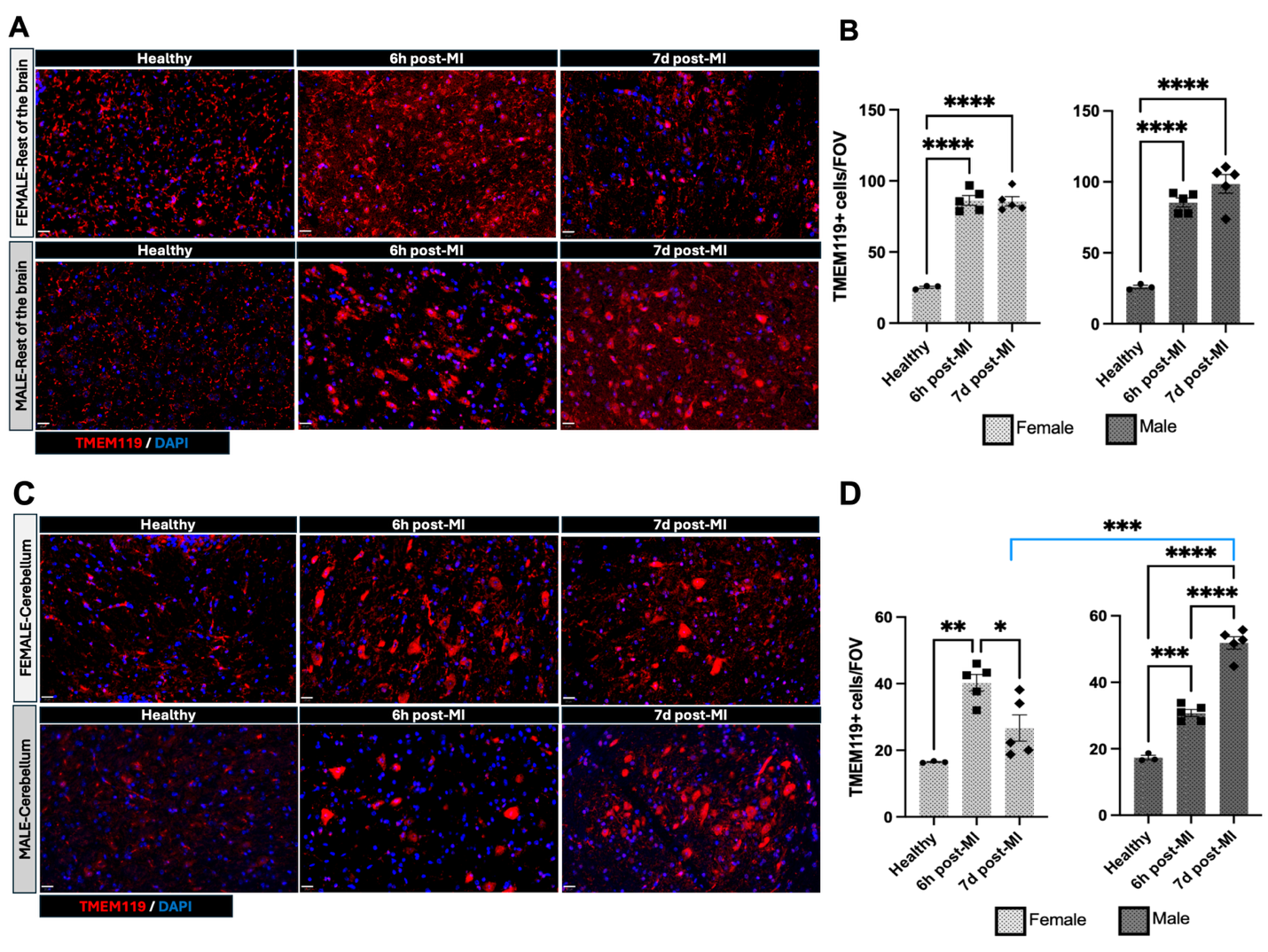


**Figure S6.** Activated microglia increase in rest of the brain and cerebellum post-MI. A) Representative immunofluorescence images of TMEM119 staining in the rest of the brain, scale bar = 20 μm. B) Quantification of the number of TMEM119^+^ activated microglia per field-of-view. C) Representative immunofluorescence images of TMEM119 staining in the cerebellum, scale bar = 50 μm. D) Quantification of the number of TMEM119^+^ activated microglia per field-of-view. Data are presented as mean ± SEM (*n* = 3 for healthy and *n* = 5 for 6h and 7d post-MI groups). Differences between groups were determined by a one-way ANOVA (black line) and differences between sexes were determined by a two-way ANOVA (blue line). ***p* < 0.01, ****p* < 0.001, *****p* < 0.0001.


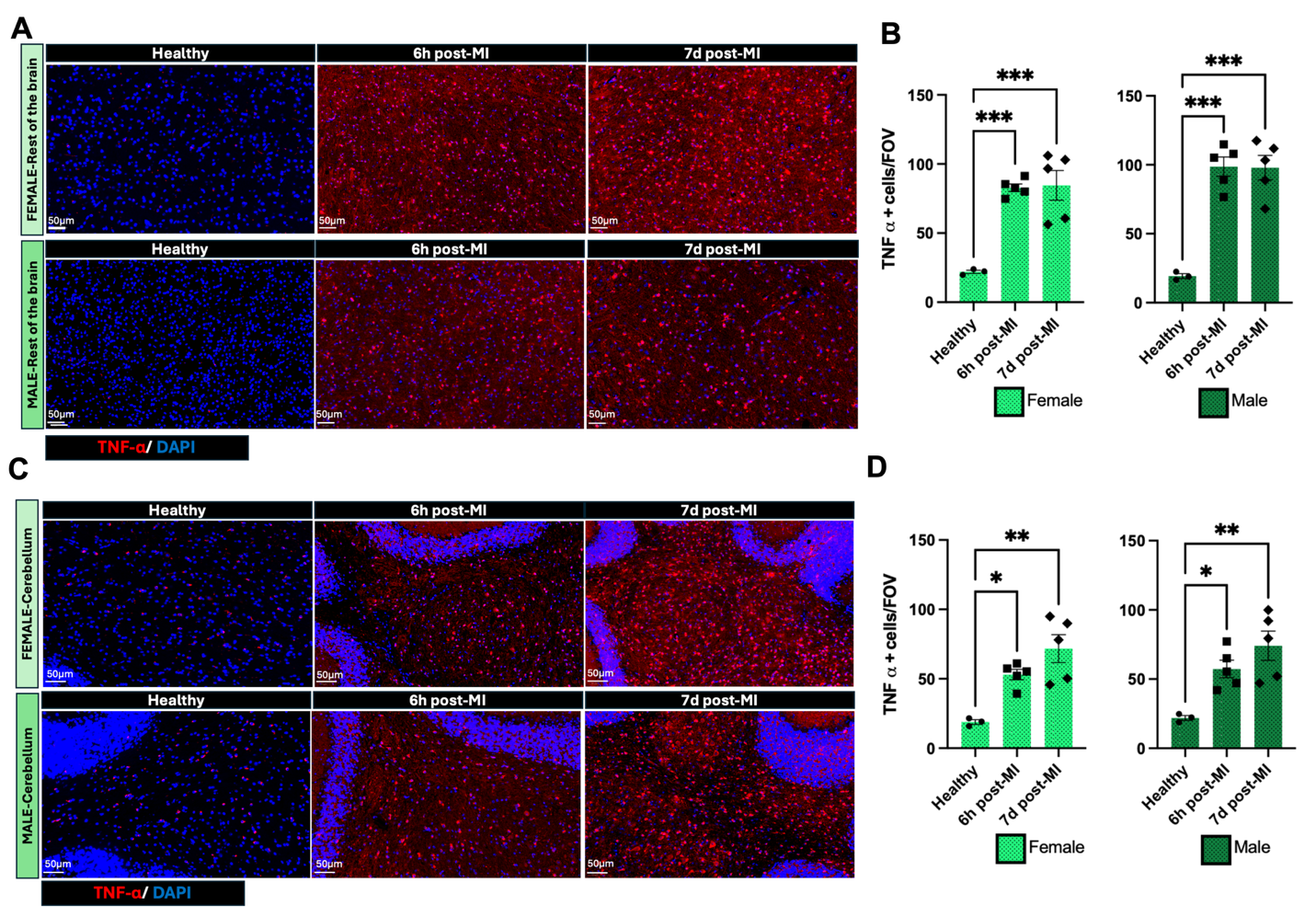


**Figure S7.** TNF-α levels increase in rest of the brain and cerebellum post-MI. A) Representative immunofluorescence images of TNF-α staining in the rest of the brain, scale bar = 50 μm. B) Quantification of the number of TNF-α^+^ cells per field-of-view. C) Representative immunofluorescence images of TNF-α staining in the cerebellum, scale bar = 50 μm. D) Quantification of the number of TNF-α^+^ cells per field-of-view. Data are presented as mean ± SEM (*n* = 3 for healthy and *n* = 5 for 6h and 7d post-MI groups). Differences between groups were determined by a one-way ANOVA (black line) and differences between sexes were determined by a two-way ANOVA (blue line). ***p* < 0.01, ****p* < 0.001, *****p* < 0.0001.


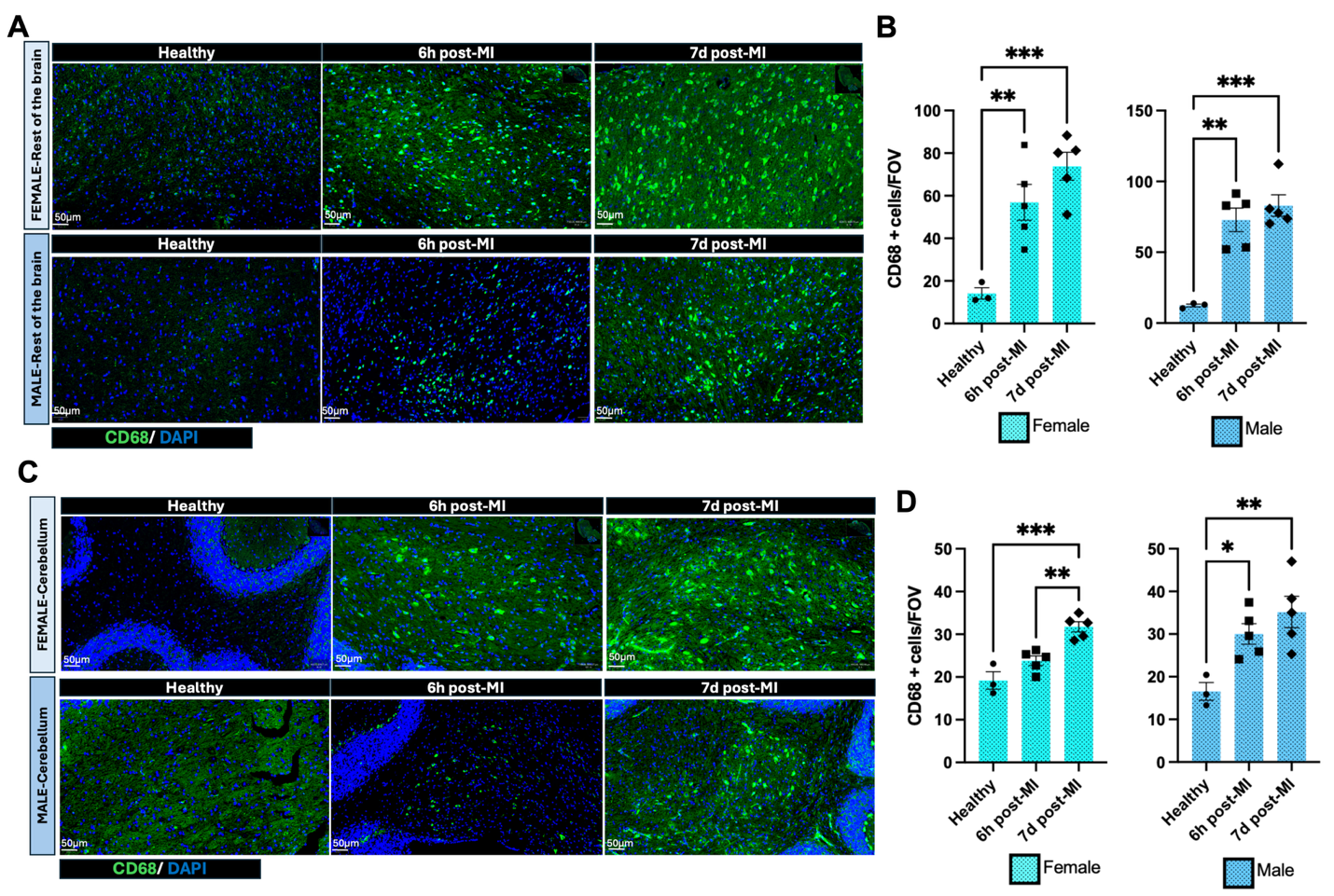


**Figure S8.** Macrophage numbers increase in rest of the brain and cerebellum post-MI. A) Representative immunofluorescence images of CD68^+^ cells in the rest of the brain, scale bar = 50 μm. B) Quantification of the number of CD68^+^ cells per field-of-view. C) Representative immunofluorescence images of CD68^+^ cells in the cerebellum, scale bar = 50 μm. D) Quantification of the number of CD68^+^ cells per field-of-view. Data are presented as mean ± SEM (*n* = 3 for healthy and *n* = 5 for 6h and 7d post-MI groups). Differences between groups were determined by a one-way ANOVA (black line) and differences between sexes were determined by a two-way ANOVA (blue line). **p* < 0.05, ****p* < 0.001, *****p* < 0.0001.


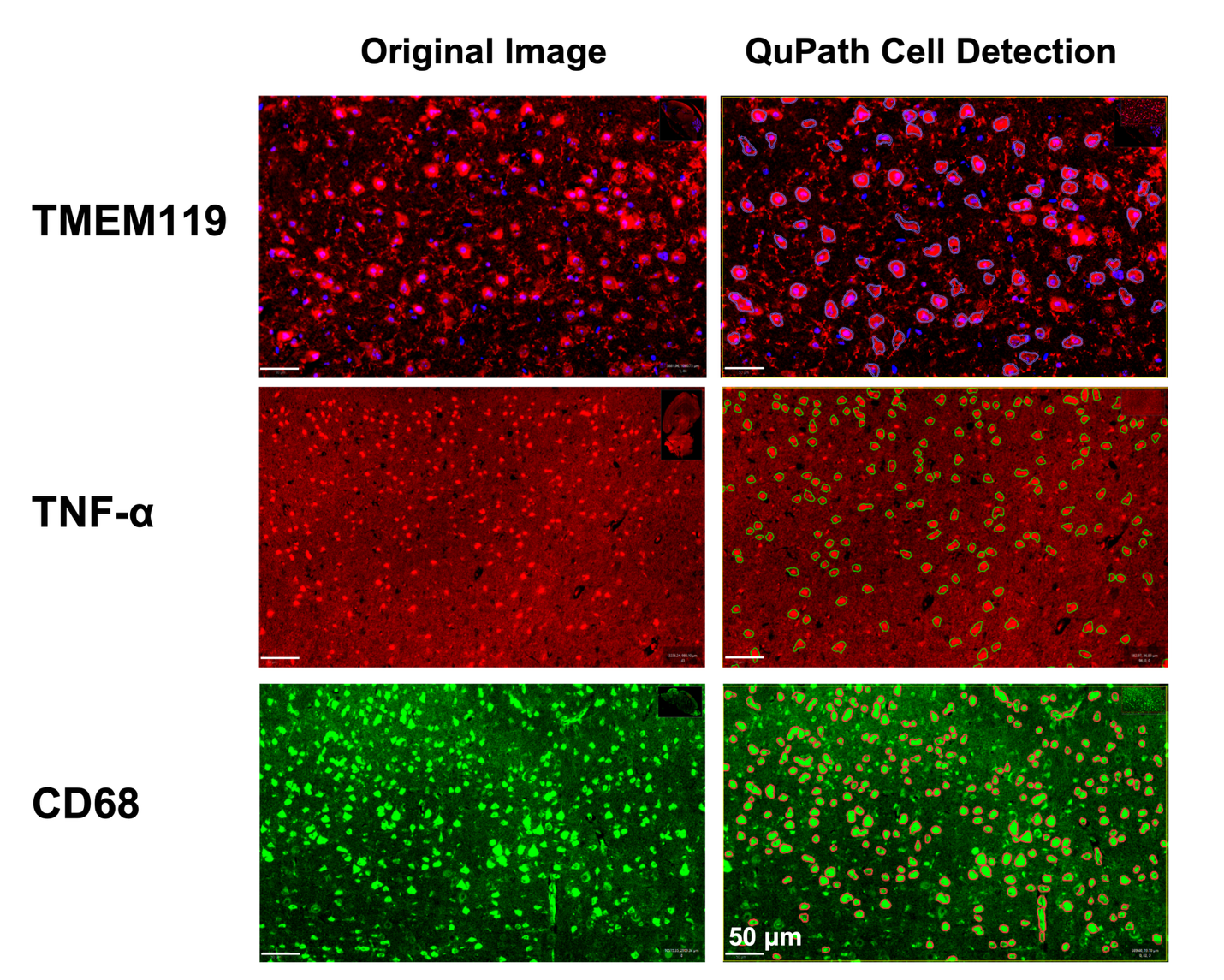


**Figure S9.** Illustration of positive cell detection using QuPath software. Scale bar = 50 μm. Created using [BioRender.com](https://www.biorender.com/).


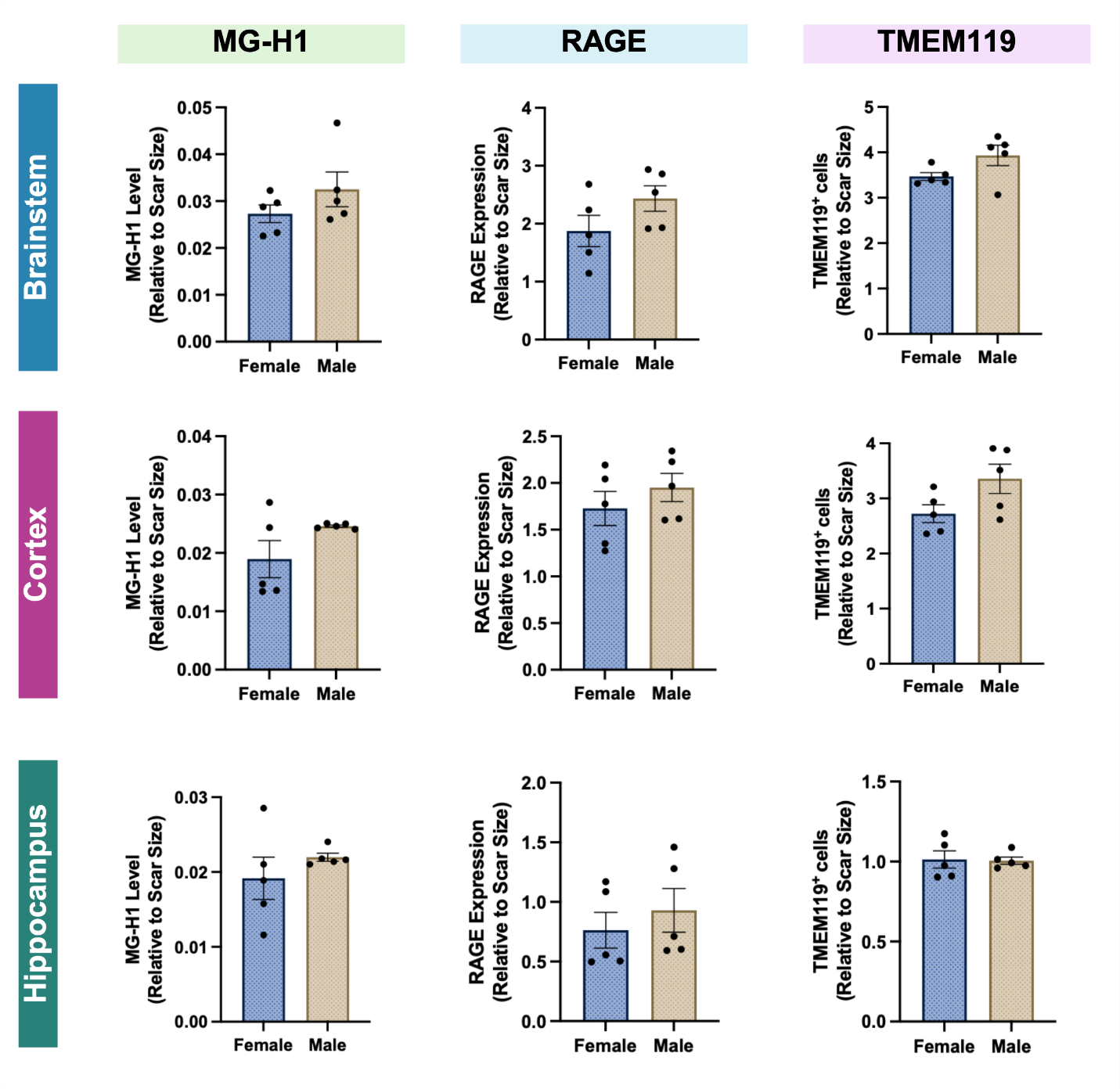


**Figure S10**. MG-H1, RAGE, and TMEM119 expression in the brainstem, cortex and hippocampus relative to scar size in female and male mice at 7 d post-MI. The calculation was performed by dividing each marker’s expression by the scar size of the same animal. Differences between groups were determined by a t-test with Welch’s correction.


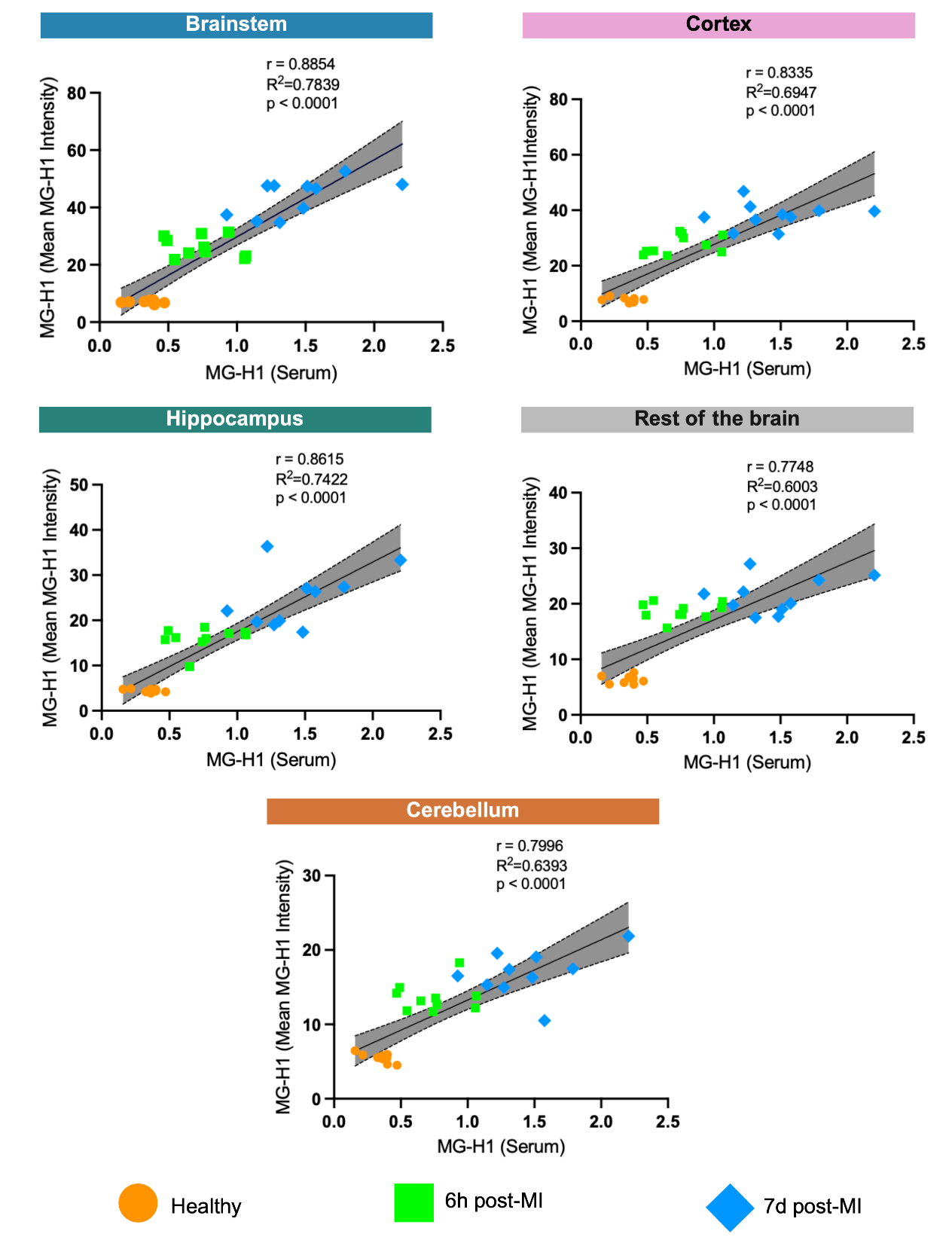


**Figure S11**. Brain MG-H1 levels correlate with the amount of MG-H1 in the serum. Correlation analysis of the MG-H1 serum level vs. the MG-H1 level in the brainstem, cortex, hippocampus, cerebellum and the rest of the brain. Male and female samples for the healthy, 6h post-MI and 7d post-MI groups were used for correlation analysis. The *r* value represents the *Pearson* correlation coefficient.

**
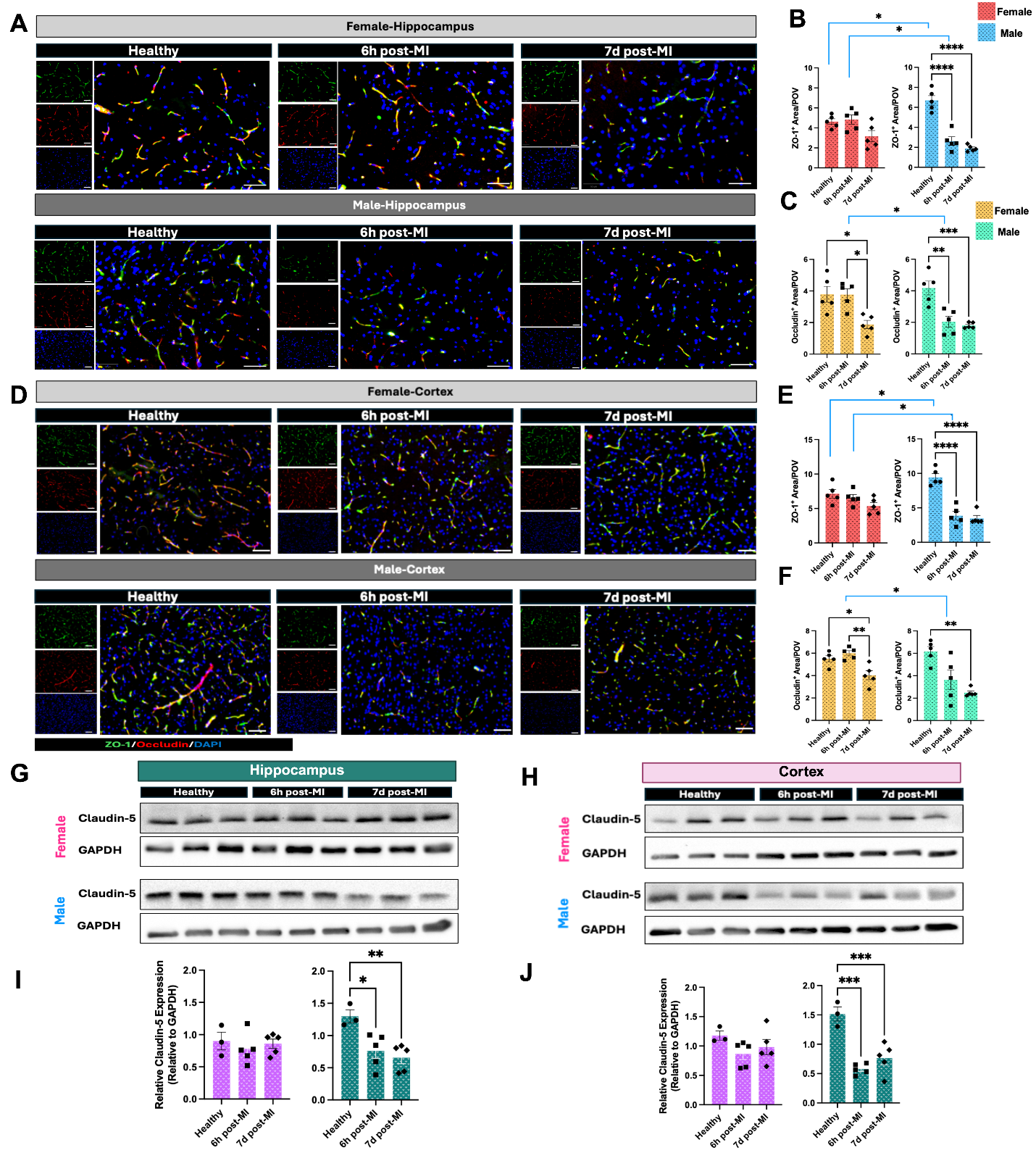
**

**Figure S12.** Tight junction proteins of the blood-brain barrier decrease in the cortex and hippocampus post-MI. A) Representative immunofluorescence images of ZO-1 and occludin staining in the hippocampus, scale bar = 50 μm. B, C) Quantification of ZO-1^+^ area (B) and occludin^+^ area (C) per field-of-view. D) Representative immunofluorescence images of ZO-1 and occludin staining in the cortex, scale bar = 50 μm. E, F) Quantification of ZO-1^+^ area (E) and occludin^+^ area (F) per field-of-view. G, H) Representative western blot images for claudin-5 in the hippocampus (G) and cortex (H). I, J) Quantification of claudin-5 relative to α-tubulin for the hippocampus (I) cortex (J). Data are presented as mean ± SEM (*n* = 3-5 for healthy and 6h post-MI group and, *n* = 5 for 7d post-MI group). Differences between groups were determined by a one-way ANOVA (black line) and differences between sexes were determined by a two-way ANOVA (blue line). **p* < 0.05, ***p* < 0.01, ****p* < 0.001, *****p* < 0.0001.


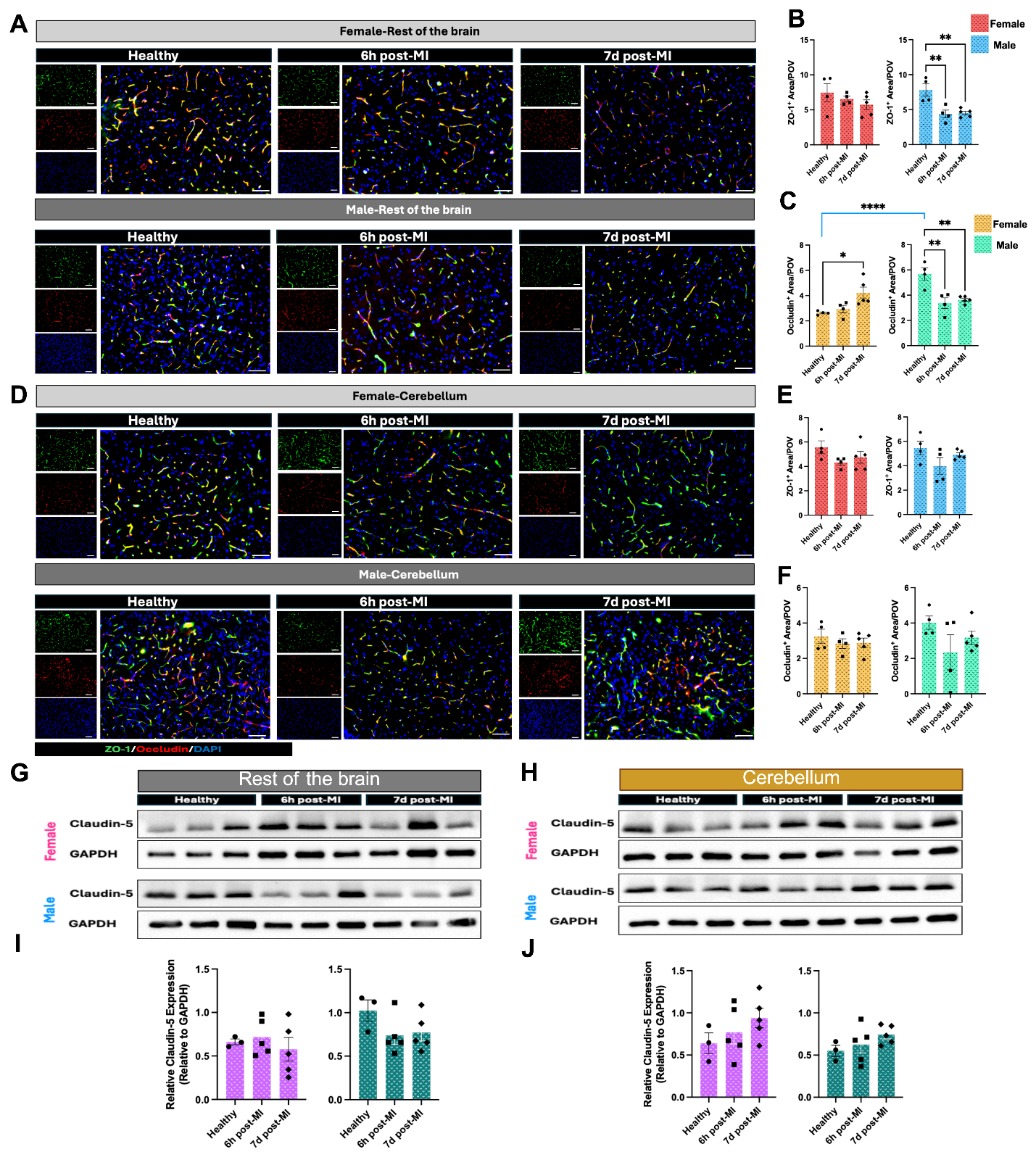


**Figure S13.** Changes in tight junction proteins of the blood-brain barrier levels in the rest of the brain and cerebellum post-MI. A) Representative immunofluorescence images of ZO-1 and occludin staining in the rest of the brain, scale bar = 50 μm. B, C) Quantification of ZO-1^+^ area (B) and occludin^+^ area (C) per field-of-view. D) Representative immunofluorescence images of ZO-1 and occludin staining in the cerebellum, scale bar = 50 μm. E, F) Quantification of ZO-1^+^ area (E) and occludin^+^ area (F) per field-of-view. G, H) Representative western blot images for claudin-5 in the rest of the brain (G) and cerebellum (H). I, J) Quantification of claudin-5 relative to α-tubulin for the rest of the brain (I) and cerebellum (J). Data are presented as mean ± SEM (*n* = 3-5 for healthy and 6h post-MI group and, *n* = 5 for 7d post-MI group). Differences between groups were determined by a one-way ANOVA (black line) and differences between sexes were determined by a two-way ANOVA (blue line). **p* < 0.05, ***p* < 0.01, ****p* < 0.001, *****p* < 0.0001.
